# Structural and mechanistic basis of anti-termination of Rho-dependent transcription termination by bacteriophage P4 capsid protein Psu

**DOI:** 10.1093/nar/gkt336

**Published:** 2013-05-22

**Authors:** Amitabh Ranjan, Savita Sharma, Ramanuj Banerjee, Udayaditya Sen, Ranjan Sen

**Affiliations:** ^1^Laboratory of Transcription Biology, Center for DNA Fingerprinting and Diagnostics, Tuljaguda complex, 4-1-714 Mozamjahi Road, Nampally, Hyderabad 500 001, India and ^2^Crystallography and Molecular Biology Division, Saha Institute of Nuclear Physics, 1/AF, Bidhannagar, Kolkata-64, India

## Abstract

The conserved bacterial transcription terminator, Rho, is a potent target for bactericidal agents. Psu, a bacteriophage P4 capsid protein, is capable of inducing anti-termination to the Rho-dependent transcription termination. Knowledge of structural and mechanistic basis of this anti-termination is required to design peptide-inhibitor(s) of Rho from Psu. Using suppressor genetics, cross-linking, protein foot-printing and FRET analyses, we describe a conserved disordered structure, encompassing 139–153 amino acids of Rho, as the primary docking site for Psu. Also a neighbouring helical structure, comprising 347–354 amino acids, lining its central channel, plays a supportive role in the Rho–Psu complex formation. Based on the crystal structure of Psu, its conformation in the capsid of the P4 phage, and its interacting regions on Rho, we build an energy-minimized structural model of the Rho:Psu complex. In this model, a V-shaped dimer of Psu interacts with the two diagonally opposite subunits of a hexameric Rho, enabling Psu to form a ‘lid’ on the central channel of the latter. We show that this configuration of Psu makes the central channel of Rho inaccessible, and it causes a mechanical impediment to its translocase activity.

## INTRODUCTION

Rho-dependent transcription termination is an essential and a well conserved process in most bacteria. Rho is a hexameric molecular motor, capable of dislodging the elongating RNA polymerase (RNAP) using its RNA-dependent adenosine triphosphatase (ATPase) activity that provides energy for its translocase function along the nascent RNA (1,2). Rho binds to the *rut* site (Rho utilization; a C-rich unstructured region) of the exiting nascent RNA, and this interaction is pre-requisite for its termination function (3). The essentiality of this protein for bacterial survival makes it a potent target for the bactericidal agents.

Rho-dependent termination induces polar effect by reducing the expressions of the downstream genes (1,2). The polarity in phage P2 is suppressed by a protein coded by *psu* (polarity suppressor), a late gene of the *Escherichia coli* phage P4 (4,5). This is a unique 21 kDa capsid-decoration protein, which was shown to moonlight as a transcription anti-terminator of the Rho-dependent termination (6–9). We had reconstituted the anti-Rho function of Psu *in vitro* and demonstrated specific complex formation between Rho and Psu (10). We also showed that Psu inhibits Rho function by affecting the latter’s adenosine triphosphate (ATP) binding, as well as the RNA-dependent ATPase activity (10). Mutational analyses showed that Psu C-terminal is important for its function, and its N-terminal is required to maintain the structural integrity (11). Recent crystal structure of Psu revealed that it is an α-helical, V-shaped knotted dimer (12) that directly binds to Rho through its C-terminal helix 7 (11).

The interacting site(s) of Psu on Rho is not known. Knowledge of the interaction surface is essential to understand the molecular basis of anti-termination. In this report, using both *in vivo* and *in vitro* techniques, we described a conserved looped out structure, encompassing 139–153 amino acids of Rho, as the primary docking site for Psu, and a neighbouring helical structure, spanning the 347–354 amino acids of Rho, plays a supportive role. Based on the conformation of Psu on the capsid structure of the P4 phage (7), its crystal structure (12), and the experimentally determined interacting regions of Rho and Psu, we have built a structural model of a Rho (hexamer): Psu (dimer) complex. In this model, a dimer of Psu forms a ‘lid’ on the central channel of the hexameric Rho. Finally, in accordance with the model, we demonstrate that binding of Psu to Rho renders the central channel of the latter inaccessible and imparts mechanical impediment to its translocase activity.

## MATERIALS AND METHODS

### Materials

NTPs were purchased from GE Healthcare. [γ-^32^P]ATP (3000 Ci/mmol), and [α-^32^P] CTP (3000 Ci/ mmol) was obtained from Jonaki, BRIT (Hyderabad, India). Antibiotics, IPTG, lysozyme, DTT and bovine serum albumin (BSA) were obtained from USB. Restriction endonucleases, polynucleotide kinase and T4 DNA ligase were obtained from New England Biolaboratories. WT *E. coli* RNA polymerase holoenzyme was purchased from Epicenter Biotechnologies. Streptavidin-coated magnetic beads were purchased from Promega. Taq DNA polymerase was obtained from Roche Applied Science. Site-directed mutagenesis kit was obtained from Stratagene. Ni-NTA agarose beads were from Qiagen and SIGMA. Details of the bacterial strains, plasmids and oligos are described in Supplementary Table S1.

### Screening for the suppressor of C-terminal Psu mutants in Rho

The strain RS659 (*rho::kan*), carrying an IPTG-dependent shelter plasmid (pHYD 1201) expressing WT *rho*, was transformed with a second plasmid pNL150 expressing different C-terminal mutants of Psu. All these *psu* were expressed from IPTG-controlled P*_tac_* promoter. Resulting strains were electroporated with a mutagenized plasmid library carrying the *rho* [pHYD567 (13,14)]. During this process, the shelter plasmid was removed by withdrawing IPTG from the media. The transformants were at first streaked (patched) on LB plate to make a master plate. Then replica plating has been done from the master plate on different LB plates containing 50 and 100 µM of IPTG. The colonies that did not grow on the IPTG plates were selected. Rho mutant plasmids were isolated from these colonies, and the suppressor mutations were confirmed by sequencing.

### Preparation of other Rho mutants

Rho mutants like R144E, R146E, E148R, R149E, K352A and V354N were made on a low copy pCL1920 plasmid by site-directed mutagenesis using the Stratagene kit. Deletion derivatives of Rho were made by overlap polymerase chain reaction (PCR) methods (15).

### Single Cys derivatives of Psu and Rho

All the single cysteine derivatives of Rho and Psu were prepared by site-directed mutagenesis, after removing their natural cysteines (two from Psu and one from Rho) by introducing C13S and C117S mutations in Psu and C202S in Rho. The single Cys derivatives of Rho were prepared on P167L Rho, instead of WT Rho. All these derivatives were active in Rho–Psu complex formation as was evident from the pull-down assays (Supplementary Figure S3A and B).

### Growth assays

**Figure 1.**
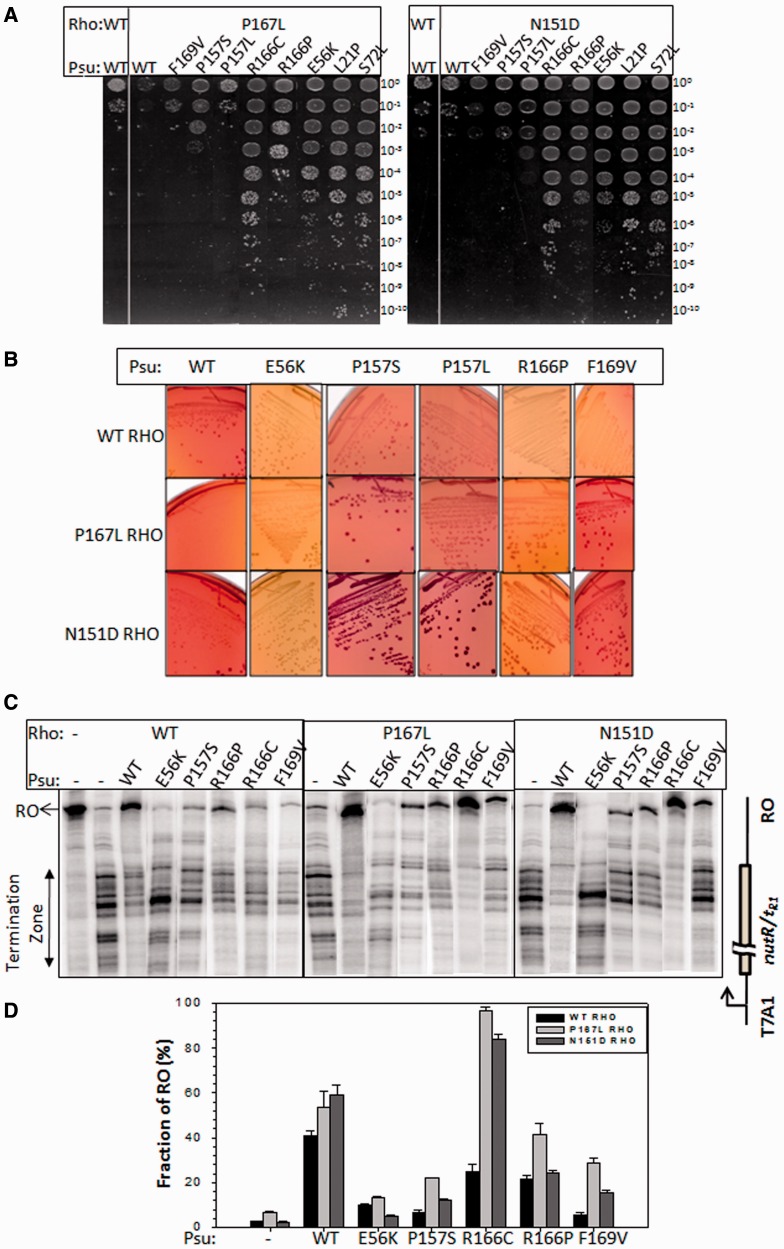
Suppressors in Rho. (**A**) MG1655*Δrho* strains (RS659) expressing WT, or P167L, or N151D Rho from plasmids were transformed with a second plasmid having WT and different Psu derivatives. Different dilutions of the overnight culture of the strains were spotted on LB plates supplemented with 100 µM IPTG to check the growth defects. Dilutions are stated by the side of the figures, which is indicative of the platting efficiencies of the different mutants. (**B**) MC4100 *galEP3Δrho* strains expressing WT or P167L or N151D *rho* from a plasmid were transformed with a second plasmid having WT and different Psu derivatives. Single colonies were streaked onto MacConkey-galactose plates. *galEP3* reporter produced red/pink colonies when Rho function was inhibited. (**C**) Autoradiogram showing the *in vitro* Rho-dependent transcription termination assays of the WT and the two Rho suppressors in the presence and absence of WT and different Psu mutants. The run-off (RO) product gives the measure of Psu-mediated anti-termination. The Rho-mediated termination zone is indicated. Concentrations of RNAP, Rho and Psu were 25 nM, 50 nM and 2 µM, respectively. (**D**) To quantitate the anti-termination activities of different Psu derivatives, the amount of RO product was plotted as bar diagrams. RO(%) was calculated as: [RO]/{[RO] + [terminated product]}. Error calculations were obtained from at least three measurements.

The strain RS659 was transformed with plasmids (pCL1920) carrying WT and mutant *rho* and plated on the LB plate in the absence of IPTG. After removing the shelter plasmid, these strains were transformed with the plasmids carrying WT and mutant *psu* (derivatives of pNL150). Serial dilutions of the overnight cultures of each of the strains were spotted onto LB plates supplemented with different concentrations of IPTG (Figure 1A), and they were grown overnight at 37°C. For the point mutants in different Psu-binding domains of Rho, instead of serial dilutions, growth assays were performed by streaking on the appropriate plates (Figure 5).

### *In vivo* pull-down assays

**Figure 2.**
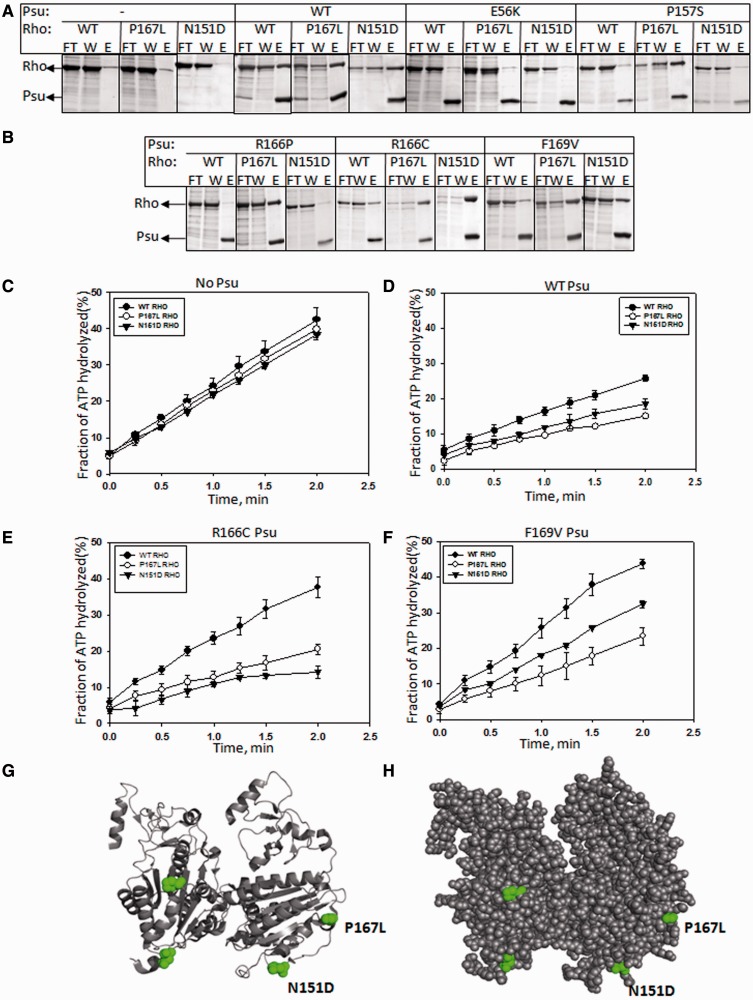
Psu binding and ATPase activities of the Rho suppressors. (**A** and **B**) *In vivo* complex formation between different derivatives of Rho and Psu proteins. Both His-tagged Psu and non–His-tagged Rho proteins were overexpressed from two different plasmids, and the cell-lysates were passed through the Ni-NTA beads. Flow through (FT) and wash (W) fractions contain the unbound Rho, whereas amount of protein in elute (E) fraction gave the measure of Psu-bound Rho. Both the proteins bands are indicated. (**C–F**) Amounts of ATP hydrolysis by WT and the two Rho suppressors, both in the absence and presence of different Psu derivatives, are plotted against time. Production of γ^32-^P from [γ^32^-P] ATP was monitored on the TLC plates. Amounts were calculated from the phosphorimager scans. Error bars were obtained from three measurements. The *λcro* RNA used to induce ATPase function was prepared by using *in vitro* transcription kit. Locations of the suppressor mutations on the structure of Rho dimer, (**G**) ribbon diagram and (**H**) space-fill model.

BL21 (DE3) strain was co-transformed with the plasmids pET28 (Kan^R^), expressing Psu proteins, and pET21b (Amp^R^), expressing Rho proteins. Psu proteins were His-tagged at the N-terminus. Transformants were inoculated in 5 ml of LB and were grown at 37°C for ∼3 h. This 5 ml culture was then added to 100 ml of LB and grown until OD_600_ ∼0.3, following which 0.1 mM IPTG was added to induce the protein expressions, and the induction was continued for 3 h. The cells were then lysed in lysis buffer (100 mM NaH_2_PO_4_, 100 mM NaCl, 10 mM imidazole, 1 mg/ml of lysozyme and 10 μg/ml of PMSF). The lysate was passed through Ni-NTA (Qiagen) columns, washed with wash buffer (100 mM NaH_2_PO_4_, 100 mM NaCl and 20 mM imidazole), and proteins were eluted with elution buffer (100 mM NaH_2_PO_4_, 100 mM NaCl and 500 mM imidazole). The volumes of lysate, wash buffer and elution buffers were kept the same for loading of the same amount of proteins in each lane (Figures 2 and 5).

### ATPase assays

The rates of ATP hydrolysis of WT and mutant Rho proteins in the absence and presence of WT and mutant Psu proteins were measured. H-19B *cro* RNA was used as an inducer. DNA templates for transcribing the RNA were prepared by PCR amplification from the plasmid pRS18 using the RS421/RK23B oligo pair. The RNA was made using the Mega transcript kit from Ambion. The ATP hydrolysis was assayed by monitoring the release of Pi from ATP using polyethylenimine thin-layer chromatography (TLC) plates and 0.75 M KH_2_PO_4_, pH 3.5, as a mobile phase under the following conditions. The assays were performed in T buffer (25 mM Tris–HCl, pH 8.0, 50 mM KCl, 5 mM MgCl_2_, 1 mM dithiothreitol and 0.1 mg/ml BSA) at 37°C. The rate of ATP hydrolysis of 100 µM of ATP together with [γ-^32^P]ATP (3500 Ci/mmol; BRIT, India) were measured using 50 nM of Rho and 5 µM of Psu, respectively. Reaction was started by addition of 0.2 µM of RNA. Aliquots were removed, and the reactions were stopped with 1.5 M formic acid at various time points depending on the concentrations of ATP. Release of Pi was analysed by exposing the TLC sheets to a Phosphor-Imager screen and subsequently by scanning using Typhoon 9200 (GE healthcare) (Figure 2).

### *In vivo* Rho-dependent transcription termination assays

We have used two reporter systems for the *in vivo* Rho-dependent termination assays. They are *galEP3* reporter, where a series of terminators are inserted in the form of *IS2* element at the beginning of the galactose operon (Figure 1B), and *lacZ* reporter system, where a double terminator cassette, *H-19Bt_R__1_-trpt',* was fused just upstream of the *lacZYA* operon (Figure 5D).

The effect of the Rho suppressors was observed to be more prominent on the *galEP3* reporter system compared with the *lacZ* system; therefore, we restricted ourselves to a less quantitative indicator plate assay (Figure 1B). These experiments were performed in the strain RS336 containing the *galEP3* reporter cassette. This strain was first transformed with the pCL1920 plasmid carrying WT and suppressor *rho* genes. Resulting strains were further transformed with pNL150 plasmids with WT and mutant *psu* genes. The transformants were streaked on MacConkey-glactose indicator plate and incubated at 37°C till the colour was developed (Figure 1).

For other point and deletion derivatives of Rho (Figure 5), the strain RS1128, containing the *H-19Bt_R__1_-trpt'-LacZ* reporter, was first transformed with the pCL1920 plasmid carrying WT and mutant *rho* genes and was subsequently transformed with the plasmid pBAD18, with or without WT and mutant *psu*. The β-galactosidase activities from the *lacZYA* reporter fused downstream of a double terminator cassette, *H-19B t_R__1_-trpt’*, give the measure of termination efficiency at these two Rho-dependent terminators. Different strains were grown until OD_600_ ∼0.2 and were then induced with 0.001% arabinose for 1 h. This level of induction was optimum for the measurement of β-galactosidase activities. Measurements were performed in a microtiter plate using Spectramax Plus plate reader [Figure 5 (13,16)].

### *In vitro* transcription assays

The T7A1-*λt_R__1_* template used for all the transcription assays was PCR-amplified from the plasmid pRS604 using the primers RS58 and RS147. Transcription on this template is initiated from the strong T7A1 promoter and proceeds through the Rho-dependent terminator *λt_R__1_*. The reactions were initiated in transcription buffer (25 mM Tris–HCl, pH 8.0, 5 mM MgCl_2_, 50 mM KCl, 1 mM DTT and 0.1 mg/ml of BSA) with 10 nM DNA template, 25 nM WT RNA polymerase, 175 μM ApU, 5 μM each of GTP and ATP and 2.5 μM CTP to make a 23mer elongation complex (EC_23_). [α-^32^P]CTP (3000 Ci/mmol; BRIT, India) was added to the reaction to label the EC_23_. This EC was then chased with 250 μM NTPs for 10 min. When required, chasing was performed in the presence of 50 nM WT or mutant Rho proteins. To see the effects of WT and Psu mutants on the Rho-dependent termination, different concentrations of WT and mutant Psu proteins were also added as indicated in the figures (Figures 1 and 5).

### Proteolytic cleavage of Rho by Fe-BABE-conjugated Psu

The single cysteine derivatives of Psu (S80C, S161C and S181C) were dialysed overnight against metal removal buffer [30 mM MOPS and 4 mM ethylenediaminetetraacetic acid (EDTA), pH 8.2], and subsequently, the protein samples were dialysed against Fe-BABE conjugation buffer (50 mM MOPS, 100 mM NaCl, 1 mM EDTA, 5% glycerol, pH 8.2). After dialysis, proteins were incubated with 5-fold molar excess of Fe-BABE (Pierce) at 37°C, in dark for 1 h. After conjugation, excess reagent was removed by passing the reaction mixture through a protein-desalting column (Pierce) equilibrated with Fe-BABE protein cutting buffer (50 mM MOPS, 120 mM NaCl, 0.1 mM EDTA, 10 mM MgCl_2_ and 10% glycerol, pH 8.0). ^32^P-labelled, 0.2 µM P167L Rho [in the heart muscle protein kinase (HMK)-tag at its C-terminal] and 2.5 µM Fe-BABE-labelled Psu were incubated at 37°C for 30 min in the Fe-BABE protein cutting buffer. The cleavage reaction was initiated by adding 4 mM each of ascorbate and hydrogen peroxide. Reaction was stopped by adding 6× sodium dodecyl sulphate (SDS) sample buffer. Samples were heated to 95°C for 4 min before loading onto 12% SDS–polyacrylamide gel electrophoresis (PAGE). To identify the positions of cleavage on Rho, molecular weight markers of the end-labelled Rho were generated by cleavage with Cyanogen Bromide (CNBr) and the proteases Arg-C and Lys-C.

### Limited tryptic digestion of Rho in the presence of Psu

C-terminal, HMK-tagged P167L Rho protein was radiolabelled with [γ-^32^P] ATP (3000 Ci/mmol) using protein kinase A. The labelling reaction was done in a buffer containing 20 mM Tris–HCl (pH 8.0), 150 mM NaCl, 10 mM MgCl_2_ and 10 μM ATP. After labelling, 0.2 μM of P167L Rho was incubated with 2.5 μM of WT Psu in transcription buffer supplemented with 1 mM ATP for 10 min at 37°C. This mixture was incubated with increasing concentrations of trypsin for 1 min. The reactions were stopped by SDS-loading dyes, and samples were heated to 95°C for 3 min before loading onto a 12–18% gradient SDS–PAGE. Molecular weight markers of end-labelled Rho were generated in the same way as described earlier in the text to identify the cleavage products.

### Rho–Psu cross-linking by a bi-functional cross-linker

We have used a bi-functional cross-linker, sulpho-LC-SPDP (sulphosuccinimidyl 6-[3′ (2-pyridyldithio)-propionamido] hexanoate (Pierce), which cross-links cysteine to amine. We first end-labelled an HMK-tagged non-cysteine derivative of Psu (C13S and C117S) with [α-^32^P]ATP (∼3000 Ci/mmol). The primary amines (lysines) of Psu were then labelled with SPDP by mixing 10 µM Psu and 30 µM SPDP in phosphate buffer (100 mM NaH_2_PO_4_, 150 mM NaCl and 1 mM EDTA, pH 7.5) for 30 min at 25°C. Excess SPDP was removed by passing the mixture through a protein-desalting column (Pierce). The SPDP-derivatized Psu was then added to different single-Cys derivatives of Rho and incubated for 10 min at 25°C in phosphate buffer. The final concentrations of Psu and Rho were ∼0.5 and ∼2 μM, respectively (see also the reaction scheme in Supplementary Figure S4A). Reactions were stopped by non-reducing SDS-sample dye (without β-mercaptaoethanol), and loaded onto a non-reducing 12% PAGE (Figure 4).

The single cysteine derivatives of Rho used in the aforementioned experiments were constructed on the P167L Rho by site-directed mutagenesis. These derivatives of Rho bind Psu efficiently (Supplementary Figure S3B).

### Fluorescence spectroscopy

All the fluorescence experiments were performed in the buffer containing 10 mM Tris–HCl (pH 7.0) and 100 mM KCl at 25°C in a Hitachi F7000 fluorescence spectrophotometer, under CAT mode, with a PMT voltage of 700 V and scan speed of 240 nm.

Excitation of Tb-GTP (3:1; 300 μM of terbium chloride and 100 μM of GTP) complex at 295 nm yields two emission peaks at 488 and 547 nm. The fluorescence intensity and polarization were monitored at 547 nm under different conditions. Fluorescence quenching was measured using a neutral quencher, acrylamide (Sigma). Change in fluorescence intensity at 547 nm was plotted against increasing concentrations of acrylamide. The quenching constant (K_SV_) was obtained using Stern–Volmer equation: (F_0_/F)_547_ = 1 + K_SV_[Q], where, F_0_ is the initial fluorescence intensity and Q is the concentration of acrylamide (Figure 7).

### FRET assays

The different single Cys derivatives of Rho and Psu proteins were dialysed against the phosphate buffer [20 mM sodium phosphate (pH 7.2), 150 mM NaCl and 8 mM EDTA]. These were then incubated with freshly prepared tetramethylrhodamine-5-maleimide (TMRA) and fluorescein-5-maleimide, respectively, (dye/protein 1:1) at 22°C for 15 min. It was followed by incubation at 4°C in the dark for overnight. The overnight incubation was done in a thermo-mixer with the interval mixing of 15 s at 350 rpm, with a pause of 5 min in between. Then the excess dye from the protein mixture was removed by 35% ammonium sulphate (35%) precipitation. The sample was centrifuged at 14 000 rpm for 1 h. The supernatant was discarded, and pellet was dissolved in the desalting buffer (Tris 10 mM, pH 8.0, KCl 100 mM and EDTA 0.1 mM) and kept for dialysis against the same buffer for 7–8 h. If free dye was still present, the proteins were also passed through Amicon ultra filters. Glycerol was added to a final concentration of 10%, and the proteins were stored at −20°C.

All of the FRET experiments were performed in a buffer containing 25 mM Tris–HCl (pH 8.0), 50 mM KCl and 5 mM MgCl_2_ at 25°C in a Hitachi F-7000 spectrophotometer. Fluorescein-labelled Psu was excited at 470 nm, and the emission spectra were recorded for 500–650 nm. Psu and the TMRA-labelled Rho proteins were mixed and incubated at 25°C for 15 min in the cuvettes before the recording of each spectrum. Concentrations of Psu and Rho proteins were 50 and 100 nM, respectively.

### Molecular docking of Psu onto the Rho

For the Rho–Psu docking, the crystal structures of the Psu dimer (PDB: 3RX6) and the Rho hexamer (PDB: 3ICE) were first modified by removing the ligands and the water molecules. The docking of the Psu dimer on the Rho hexamer was performed using the program ‘O’ (17) that is based on the constraints from the previous experimental observations, results of the biochemical experiments of this study and maintaining a symmetric interaction at both the binding sites. The docked model so obtained was refined using the docking refinement module in Nomad-Ref server (18) using the default parameters. The binding free energy of the refined docked model was calculated using PISA (19).

### RNA:DNA hybrid unwinding assays

The DNA template for this assay was made by PCR amplification on the plasmid pRS106 (T7A1-*trpt’-lacZYA*) using biotinylated primer RS83. A lac operator sequence was inserted at the position +161 by using a downstream primer having this sequence. The template (T7A1-*trpt'-lacO*) was immobilized on the streptavidin-coated magnetic beads (Promega) via the 5′-end biotinylation. Transcription reactions were initiated with 30 nM template, and the EC_23_ complex was made in a similar way as in other *in vitro* transcription reactions (Figures 2 and 5), except that 100 nM Lac repressor was added. EC_23_ was then chased in the presence of 200 μM NTPs and rifampicin (10 μg/ml) for 2 min to make a stalled elongation complex (RB) at the lac repressor site. The RB was washed thoroughly to remove NTPs. RNA:DNA hybrid was formed by adding 10 μM of antisense oligo (antisense to the *rutB* site, 20 nt upstream of the 3′-end of the nascent RNA in the RB; 5′-GAGGAATAAGTGACTTAGAG) to the nascent transcript. This was followed by addition of 50 nM WT Rho either in the absence or presence of WT or F169V Psu proteins. The Rho was activated by adding 1 mM ATP along with 10 μM of the sink-oligo (complementary to the antisense *rutB* oligo, 5′-CTCTAAGTCACTTATTCCTCAGG), to ensure a single-round unwinding reaction. The reaction was incubated at 37°C for 5 min followed by addition of 0.05 U/μl of RNase H. The RNase H digestion was allowed for 1 min before the reaction was stopped by addition of phenol. The RNA was phenol extracted and loaded onto a 10% denaturing sequencing gel to fractionate the products.

## RESULTS

### Isolation of suppressors in Rho

In an earlier report, we have characterized several point mutations in Psu that are defective for Rho binding and established that its C-terminal region (170–190 amino acids) directly interacts with Rho (11, see Supplementary Figure S1A for the mutations). Hence, to get an indication of the Psu-interacting region(s) of Rho, we looked for the allele-specific suppressors in Rho for the C-terminal Psu mutants, P157L/S, R166C/P and F169V. An MG1655 strain (RS659) with a chromosomal deletion of *rho* (*rho::kan*), carrying the plasmid pNL150 and expressing any one of the aforementioned Psu C-terminal mutants from an IPTG-controlled *P_tac_* promoter, was transformed with a low-copy pCL1920 plasmid having a mutagenized library of *rho* expressed constitutively from its own promoter. Transformants were at first obtained on LB plates, and then each of them was replica platted on LB plates with different concentrations of IPTG (see ‘Materials and Methods’ section for details). The expression of *psu* is minimal on the LB plates, whereas it is higher on the LB + IPTG plates. High-level expression of WT Psu proteins inhibit WT Rho function and inflict lethality to the cells (10), whereas the aforementioned C-terminal Psu mutants can not cause this defect (11). On the replica plates (i.e. in the presence of IPTG), we looked for *rho* mutants from the mutagenized library that caused lethality in the presence of *psu* mutants. We obtained two such Rho mutants, N151D and P167L that were lethal with the Psu mutants, F169V and P157L, respectively.

We checked the growth phenotypes of different *psu* mutants in the presence of these two *rho* mutants (Figure 1A). Growth defects were estimated by spotting different dilutions of overnight cultures having different combinations of Rho and Psu mutants. Lack of cell growth at lower dilutions indicates the Psu-induced lethality. Consistent with our previous observations (10,11), WT Psu/WT Rho combination was lethal (leftmost column of Figure 1A). N151D Rho specifically suppressed the growth defects caused by F169V, P157S and P157L Psu mutants, whereas the same defect caused by P157S, P157L, R166P and F169V mutants of Psu was suppressed by P167L Rho. They specifically suppressed the defects of C-terminal mutants of Psu and not the growth defects of N-terminal ones (E56K, L21P and S72L columns; Figure 1A).

### Rho suppressors overcome the anti-termination defects of the Psu mutants

Next we tested whether these two Rho mutants can overcome the anti-termination defects of the C-terminal Psu mutants. For the *in vivo* anti-termination assays, we have used MC4100 strain having a single-copy *galEP3* reporter (see ‘Materials and Methods’ section) in the chromosome. This reporter cassette consists of a series of Rho-dependent terminators present in the *IS2* element inserted at the beginning of the galactose operon. In the presence of functional Psu, Rho is inhibited, and RNAP overcomes these terminators to express downstream genes of the galactose operon. Expression of these genes make the strain capable of using galactose from the media that renders the formation of pink/red colonies on the MacConkey-galactose plates. We observed that, WT and E56K Psu proteins induced red and white colonies, respectively, with all the Rho derivatives (Figure 1B). Strains with the C-terminal mutants of Psu appeared as white, when WT Rho was present. The strains expressing P157S and F169V Psu proteins became red/pink in the presence of P167L Rho. Also the strains with P157S, P157L and F169V Psu appeared as red/pink when N151D Rho was present. Defect because of the R166P Psu was partially suppressed by these two Rho derivatives. These results correlate with the suppression of growth defects shown in Figure 1A.

The effect of the Rho suppressors were then tested *in vitro* transcription assays. We have used a linear DNA template, where transcription is initiated from the strong T7A1 promoter, and in the presence of WT Rho, it terminates over a zone of a Rho-dependent terminator, *λt_R__1_*. Under our experimental conditions, this terminator functioned efficiently in the presence of WT Rho (%RT <10%, see Figure 1D, height of the bars under −Psu conditions). The amount of run-off transcript (RO) indicates the Psu-induced anti-termination at this terminator. All the Psu mutants tested were defective in anti-terminating the WT Rho (Figure 1C, left and Figure 1D). Interestingly, all the C-terminal Psu mutants were able to anti-terminate P167L Rho with moderate to high efficiency (Figure 1C, middle and histograms in Figure 1D). But, N151D Rho was efficiently anti-terminated only in the presence of R166C Psu (Figure 1C, right). It is likely that because of less stringent conditions *in vitro*, the suppressor effects were less specific compared with that observed in the *in vivo* termination assays (Figure 1B).

### Rho suppressors rectify the Rho-binding defects of the Psu mutants and their defect of inhibiting ATPase activity of Rho

Earlier, we have reported a method to demonstrate the *in vivo* Rho–Psu complex formation, by overexpressing the two proteins from two separate plasmids (pET vectors) having different antibiotic markers, where Psu is histidine-tagged at its N-terminal and Rho is untagged. The cell lysate is passed over the Ni-NTA beads, and different fractions are monitored on SDS–PAGE [(10) see also ‘Materials and Methods’ section]. We co-overexpressed the WT and the two Rho suppressors together with different Psu mutants (Figure 2A and B). Amounts of WT, N151D and P167L Rho obtained in the elute (E) fractions, indicating the extent of complex formation with the Ni-NTA-bound WT and different derivatives of Psu, were estimated (Supplementary Figure S1B). We observed that P167L Rho was efficiently pulled down by WT and all the C-terminal mutants of Psu. N151D Rho demonstrated more specific binding to WT, R166C and F169V Psu mutants. None of the Rho derivatives interacted with the N-terminal Psu mutant, E56K. Compared with WT Rho, the P167L Rho seemed to have tighter binding to WT Psu. *In vivo* association of both the Rho derivates with R166C Psu was observed to be the most efficient.

These results indicated the following. (i) The gain of function in the Psu mutants, arose from their ability to interact with these two Rho suppressors. (ii) The Rho suppressors specifically bind to the C-terminal Psu mutants. (iii) P167L Rho has an enhanced affinity for the WT and all the C-terminal Psu mutants.

Binding of Psu to Rho slows down the rate of RNA-dependent ATP hydrolysis of the latter [(10) also compare the slope of the plots in Figure 2C and D]. Consistent with their lack of interaction with WT Rho, the Psu mutants, R166C and F169V, did not show significant inhibition of ATPase activity of the WT Rho (Figure 2E and F). But they regained their inhibitory activity with these two Rho suppressors (Figure 2E and F). However, the inhibitory activity was not as strong as that observed for the WT Psu on the WT Rho. These results were also consistent with the other properties of P167L and N151D Rho mutants.

Based on aforementioned *in vivo* and *in vitro* assays, we concluded that N151D and P167L mutants of Rho indeed function as suppressors of different C-terminal mutants of Psu, and this suppression was achieved by direct interaction with the Psu derivatives that led to the inhibition of the Rho functions. However, these suppressors are not strictly allele specific in nature. We localized these two suppressor mutations on the Rho structure (Figure 2G and H). They are located near the exit path of RNA emerging out of the secondary RNA-binding channel and are also surface exposed. It is possible that these or nearby region(s) of Rho could be the interaction site(s) for Psu.

### Cleavage of Rho by Fe-BABE-conjugated Psu

Next we used different protein foot-printing, cross-linking and FRET techniques to probe the interaction site(s) of Psu on the Rho protein. We observed that, unlike WT Rho, one of the suppressor, P167L Rho, has higher affinity for WT Psu, and it forms a stable complex *in vitro* (Supplementary Figure S2). Psu in this complex efficiently inhibits different functions of Rho (Figures 1 and 2). And, hence, instead of WT Rho, we used P167L Rho:WT Psu complex for all these aforementioned *in vitro* assays.

**Figure 3.**
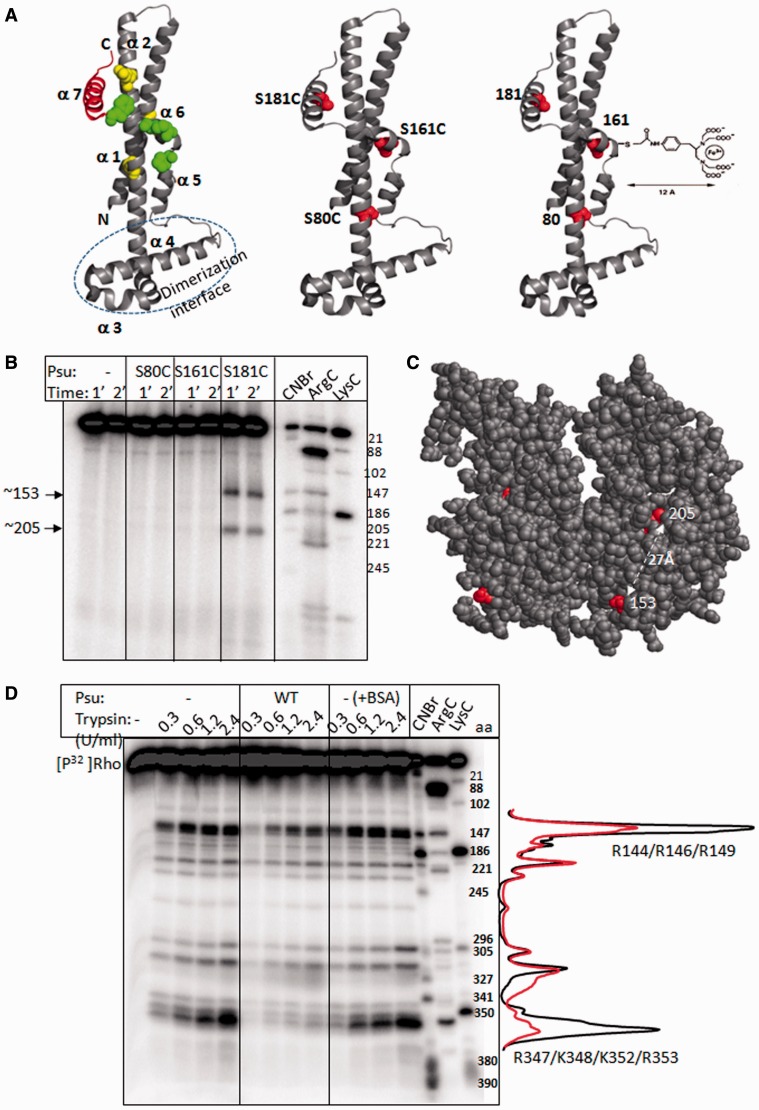
Foot-printing analyses of the Rho–Psu complex*.* (**A**) Seven α-helices and the dimerization interface on the crystal structure of Psu monomer are shown (left; PDB code: 3RX6). The locations of Psu mutants defective for inhibiting Rho are indicated. C-terminal tail region (helix 7; 170–190) is in red. C-terminal point mutations at 169, 166 and 157 positions are indicated in green spheres. N-terminal mutations at 21, 56 and 72 are shown in yellow spheres. Single Cys derivatives at positions 181, 161 and 80 of Psu are indicated in red spheres (middle). An example of the conjugation of Fe-BABE moiety to one of the Cys residues is shown in right. (**B**) Autoradiogram of the P^32^-labelled HMK-tagged P167L Rho. Fe-BABE tagged different single Cys-derivatives of Psu were added to Rho, and the Fenton reactions were continued for indicated time. The indicated cleavage positions were identified by comparing with the molecular weight markers generated by CNBr, ArgC and LysC. Amino acid positions are indicated. (**C**) The positions 153 and 205 are indicated on the structure of Rho dimer (PDB: 2HT1). (**D**) Autoradiogram of P167L Rho in the presence of different concentrations of trypsin under different conditions. The trypsin cleavage sites were identified from the size markers obtained from the proteolytic cleavages of the same Rho molecule using different proteases as indicated. Profile plots of the cleavage intensities are shown adjacent to the autoradiogram. Trypsin cleavage of Rho in the absence of Psu was conducted both in the presence and absence of BSA (the +BSA lanes).

We used the tethered·OH-generating reagent, Fe-BABE ([iron (bromoacetamidobenzyl)-ethylenediaminetetraacetate], which can be attached to Cys residues, and on activation of·OH cleaves nucleic acids and proteins present in its vicinity (20). We conjugated Fe-BABE to the single cysteines of the Psu derivatives, S181C, S161C and S80C (Figure 3A). S181C and S161C are located within or near the Rho-interaction region of Psu, whereas S80C is located away from this region (Figure 3A). These single Cys substitutions did not affect their binding to P167L Rho (Supplementary Figure S3A).

The Fe-BABE-conjugated Psu derivatives were complexed with P167L Rho in the presence of 1 mM ATP. On activation of OH-radical generation, we observed that only Fe-BABE conjugated to the 181 position of Psu produced two cleavage products near/at 153 and 205 amino acids positions of Rho (Figure 3B). This cleavage product arose due to the vicinity of this C-terminal helix 7 [marked in red, Figure 3A (12)] of Psu to Rho, and it is consistent with the functional importance of this helix in the anti-termination process (11). The cleavage site on Rho, the amino acid 153, is located close to the position of N151D suppressor. Therefore, this site may define the primary interaction site for Psu.

Fe-BABE conjugated to 161 and 80 positions of Psu did not yield any product. It is possible that because of limited degrees of freedom of the bulky Fe-BABE moiety, there was no cleavage from the position 161. The amino acid 80C is located far away from the interacting helix 7 (in red, Figure 3A); hence, lack of cleavage from this position is not unexpected.

The amino acid 205 is ∼27 Å away from the 153 position of Rho (Figure 3C). Cleavage at 205 could define a second interaction site(s) for Psu. However, OH-radical can travel ∼10 Å before getting quenched by water molecules. Therefore, from the centre of Fe-BABE moiety, this radical can cleave within a sphere with a radius of ∼22 Å (Figure 3A, right). Therefore, it is also possible to obtain a cleavage at position 205 that is located away from the primary interaction site.

### Protein foot-printing of the Rho–Psu complex

**Figure 4.**
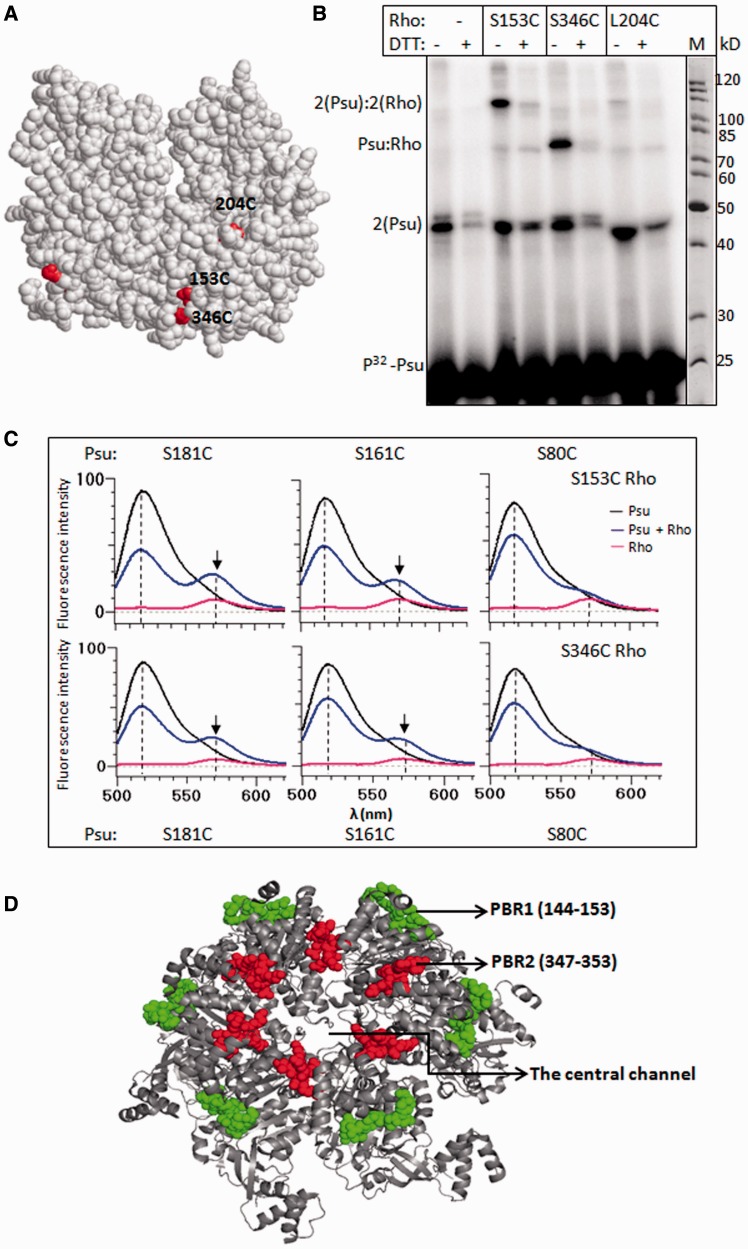
Chemical cross-linking and FRET analyses*.* (**A**) The locations of the single Cys residues that were labelled in the cross-linking and FRET assays, on the structure of Rho dimer (PDB: 2HT1). (**B**) Autoradiogram of the radiolabelled Psu. The single Cys derivatives, made on the P167L Rho, as indicated were mixed with SPDP-labelled Psu. Different cross-linked products are indicated, and their assignments were based on the molecular weight. (**C**) Fluorescence emission spectra of Psu proteins with fluorescein-labelled at different single Cys residues, in the absence and presence of different TMRA-labelled Rho proteins, are shown in different panels. Excitation is at 470 nm, and emission was monitored for 500–620 nm. The enhancement of emission at 570 nm is due to the FRET between fluorescein- and TMRA-labelled Cys residues of both the proteins. Emission intensity of TMRA–Rho in the absence of Psu is shown in each panel as control. (**D**) The two PBRs are decorated by coloured spheres on the hexameric structure of Rho (PDB code: 2HT1, 23).

Next we used protease-mediated protein foot-printing of Rho–Psu complex to identify the region(s) on Rho protected by Psu. We used limited trypsin digestion to generate the footprint (Figure 3D). We observed that the two prominent trypsin cleavage sites of Rho, located at 144–149 and 347–353 amino acids, were significantly protected in the presence of Psu. The footprint at 144–149 amino acids is consistent with the Fe-BABE cleavage at the position 153 (Figure 3B), and it is situated near the suppressor at 151 position (Figures 1 and 2). The protection at 347–353 amino acids was strong and also lies close to the 144–149 amino acids (Figure 4D). It is likely that region around 347–353 amino acids of Rho could be the second interaction site for Psu, and both the interaction sites may come even closer on interaction with Psu. The lack of trypsin cleavage at 347–353 amino acids of Rho could also arise from the Psu-induced conformational changes.

### Site-specific cross-linking between Rho and Psu

To further test the proximity of the previously described three regions of Rho to the helix-7 of Psu, we constructed three single Cys derivatives in these regions of Rho as S153C (region 139–153), L204C (region around 205) and S346C (region 347–353) (Figure 4A). These Rho derivatives formed stable complex with Psu (Supplementary Figure S3B). We induced cross-linking between each of these Cys residues of Rho, and the surface exposed lysines of Psu via a bi-functional cross-linker, LC-SPDP (sulphosuccinimidyl 6-[3′(2-pyridyldithio)-propionamido] hexanoate; Supplementary Figure S4A). The cross-linking will occur if these cysteine residues of Rho come within ∼15 Å of the surface of Psu (Supplementary Figure S4A). We observed the formation of three kinds of cross-linked species migrating around ∼45, ∼75 and ∼110 kDa, those correspond to the sizes of a Psu dimer [2(Psu)], a complex of Psu monomer and Rho monomer [Psu:Rho] and a complex between Psu dimer and Rho dimer [2(Psu):2(Rho)], respectively (Figure 4B). All these species involved −SH linkages because they were sensitive to DTT (+DTT lanes). The 153C Rho formed a [2(Psu):2(Rho)] complex with Psu, whereas 346C Rho formed a [Psu:Rho] complex. No specific cross-linked species was observed from 204C of Rho. Western blots with Rho antibody confirmed the presence of Rho in these two cross-linked species (Supplementary Figure S4B).

Two SPDP adducts from the two subunits of Psu can form di-sulphide bridges (Psu-S-S-Psu), if two lysines from each of the subunits come within 30 Å (Supplementary Figure S4C, left). If a proper geometry is achieved, as was in the case of S153C Rho, this Psu dimer could form a 2Psu:2Rho complex with Rho (Supplementary Figure S4C, right). On the other hand, because of spatial constraints, a Psu:Rho complex was formed in the presence of S346C Rho.

These results also suggest that the region around 205 amino acid of Rho does not take part in direct interaction with Psu. Cross-linking from the regions 139–153 amino acids and 347–354 amino acids is indicative of the proximity of these regions of Rho with Psu in a Rho:Psu complex that further confirmed the observations described in Figure 3.

### FRET measurements between the Cys–Cys pairs of Rho and Psu

To provide supporting evidence for these cross-linking data, we measured the efficiency of FRET between the fluorescent-labelled Cys pairs of Rho and Psu. The Cys residues of Rho were selected from the aforementioned cross-linking assays (Figure 4B), and those from Psu were chosen from the Fe-BABE cleavage assays (Figure 3B). We used the fluorophores, fluorescein (FL) and rhodamine, as the donor–acceptor pair for these assays. The distance for 50% energy transfer efficiency (the Forster radius, R_0_) for this pair is 49–54 Å (21). The single Cys derivatives of Psu, S80C, S161C and S181C, were labelled with FL, and the two single Cys Rho derivatives, S153C and S346C, with TMRA. An efficient FRET between these Cys pairs occurs only when they come within a distance range of ∼50 Å in the Rho–Psu complex. In this case, if distance constraint is satisfied, one can expect an emission around 570 nm (the emission wavelength range of Rhodamine) on excitation at 470 nm (excitation wavelength of FL).

Figure 4C showed that an enhancement of emission intensity at 570 nm occurred when the Rho:Psu complex was formed with all the Cys pairs, except in the cases where S80C Psu was present (compare the panels in Figure 4C). These results indicated an efficient FRET between 161 and 181 positions of Psu (helix 7 and loop between helices 6 and 7; see Figure 3A) with the amino acids 153C and 346C of Rho, and they further reinforced the proposition that regions surrounding 147–153 and 347–355 amino acids of Rho come close to the C-terminal helices of Psu. The FRET assays were also consistent with cross-linking and Fe-BABE cleavage assays (Figures 3B and 4B).

Therefore, results obtained from Fe-BABE cleavage, trypsin-foot-printing, SPDP–cross-linking and FRET unequivocally suggest that there are two Psu-binding regions (PBRs; PBR1 and PBR2) in Rho, and they form a lining around the central channel of the hexameric helicase (Figure 4D).

### Identification of Rho mutants defective for Psu function

Next we attempted to establish the functional significance of the PBR1 and PBR2 regions of Rho in the complex formation with Psu. We made several point mutations and deletions in these two regions, and used *in vivo* and *in vitro* binding and transcription assays similar to those described in Figures 1 and 2.

**Figure 5.**
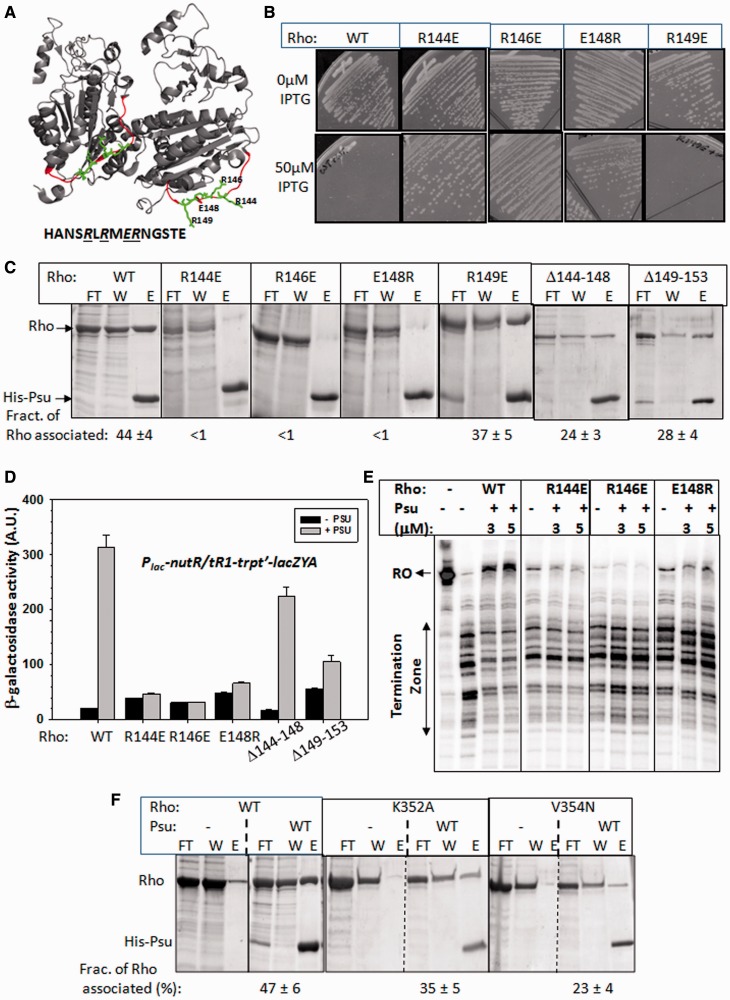
Functional importance of the PBRs. (**A**) A dimer of Rho showing the PBR1 region. The sequence of this region and the amino acids that are mutated are indicated. (**B**) MG1655*Δrho* strains expressing either WT or mutant *rho* from the plasmids were transformed with the second plasmid having WT *psu*. Strains were streaked on LB plates in the presence and absence of IPTG as indicated. (**C**) *In vivo* complex formation between different derivatives of Rho and Psu proteins. Experiments were done in the same way as in Figure 2. Flow through (FT) and wash (W) fractions contain the unbound Rho, whereas the amounts of protein in elute (E) fraction gave the measure of Psu-bound Rho. Both the proteins bands are indicated. Fraction of Rho associated was calculated as {[E]/[FT] + [W] + [E]}. (**D**) *In vivo* anti-termination assays of different Rho mutants. The values of β-galactosidase activity from the *lacZYA* reporter fused downstream of a double terminator (*t_R1_-trpt'*) cassette is plotted. Measurements were made both in the absence and presence of WT *psu* cloned in a plasmid. Error bars were obtained from the average of five to six measurements. (**E**) Autoradiogram showing the *in vitro* termination assays of the Rho mutants in the presence of indicated concentrations of WT Psu. DNA template and other experimental conditions were same as in Figure 1C. (**F**) *In vivo* complex formation assays performed with the PBR2 mutants of Rho, in a similar way as in C, having the same notations.

From the Rho structure, we identified a looped out disordered region in PBR1 (Figure 5A). We hypothesized that this loop region may act as a Psu-docking site, and the highly reactive charged amino acids in this loop could provide favourable columbic interactions. And, hence, we constructed the following point mutants: R144E, R146E, E148R and R149E. We also constructed two micro-deletions, Δ(144–148) and Δ(149–153), in the PBR1 (Figure 5A). These changes did not induce significant secondary structure changes in the Rho proteins (Supplementary Figure S5A).

We at first checked whether Psu can induce growth defects to the strains having these Rho mutants. We performed similar growth assays as described in Figure 1A, and we observed that except R149E, all the mutants showed healthy growth in the presence of Psu (Figure 5B). This suggested that Psu was unable to inhibit the functions of R144E, R146E and E148R mutants of Rho. To know whether this defect of Psu arose from its inability to bind to Rho, we monitored the *in vivo* Rho–Psu complex formation by similar pull-down assays as described in Figure 2A. All the Rho mutants, except R149E, were unable to form complexes with Psu (Figure 5C), as the amounts of Rho mutants eluted reduced to the level of their non-specific adsorption on the Ni-NTA beads (Supplementary Figure S5). However, in the same assay, the two micro-deletions in Rho showed partial binding defects. In these cases, amounts of Rho were moderately higher than their non-specific adsorption levels (Supplementary Figure S5B).

Next, we conducted *in vivo* and *in vitro* transcription termination assays. The amounts of β-galactosidase activities from a *lacZ* reporter fused downstream of a double terminator cassette, *nutR*/*tR1-trpt'*, gave the measure of the anti-termination efficiency through these terminators. In these assays, Psu-induced anti-termination increases the values of β-galactosidase activities. We observed that, unlike the WT Rho, the point mutants did not show significant anti-termination in the presence of Psu (Figure 5D). The Δ(149–153) Rho showed weak anti-termination, whereas the effect of Δ(144–148) Rho was moderate (Figure 5D). We also conducted *in vitro* transcription assays on a linear DNA template, where a Rho-dependent terminator, *λt_R__1_*, was cloned downstream of a strong T7A1 promoter. The run-off product (RO) obtained in the reaction was the measure of the anti-termination efficiency. Consistent with the *in vivo* assays, the Rho point mutants were defective for Psu-mediated anti-termination (Figure 5E).

We next tested the functional importance of PBR2 of Rho in the Rho–Psu complex formation. We constructed two point mutants in this region, K352A and V354N, which are the most surface-exposed amino acids of this region. The *in vivo* binding assays with these mutants were carried out in a similar way as in Figure 5C. The Psu-binding defect of V354N Rho was significant, whereas it was moderate for the K352A (Figure 5F). Even though these two mutations showed partial defects in the *in vivo* complex formation with Psu, this was not reflected in Psu-mediated *in vivo* anti-termination assays. Psu was also able to induce anti-termination with these two Rho mutants at the *nutR/t_R__1_-trpt'* terminator cassette (Supplementary Figure S6A). As the *in vivo* pull-down assays are not quantitative, we did not highlight this defect, and we attempted to compare between the binding and activity assays. Mutations in other two nearby positions, 385 and 397, also did not affect the Psu activity (Supplementary Figure S6B).

These results indicated the following. (i) PBR1, consisting of an unstructured looped out region, is functionally important for the interactions with Psu. (ii) Less severe effect of Δ(144–148) indicates that this part of the loop may not be interacting directly. However, presence of oppositely charged amino acids in the point mutants, R144E, R146E and E148R, might have imparted strong repelling effect on Psu. (iii) The relatively stronger effect of the Δ(149–153), together with cross-linking and Fe-BABE cleavage data strongly indicate that this part of the loop region could be the docking site of Psu. (iv) In the Rho–Psu complex, Psu may not make functional interaction with PBR2, but this region stays close to the interaction surface.

### A structural model of the Rho–Psu complex

We generated a structural model of Rho–Psu complex based on the following information. (i) The distance between the two C-terminal tail located diametrically opposite direction from the ‘V’-shaped structure of Psu dimer [(12) Supplementary Figure S7A)]. (ii) The electron microgram of a Psu dimer covering the central hole of the hexameric capsid protein, Sid [(7) Supplementary Figure S7B)]. (iii) Interacting regions defined by the genetic and biochemical data described in Figures 1–5. (iv) The Rho-interacting regions of Psu [Figure 3A; (11,12)]. (v) Inhibition of ATP binding as well as ATPase activity of Rho by Psu (10). The ATP-binding sites of the Rho hexamer (PDB code: 3ICE) are located at the inter-subunit grooves, with an average distance of ∼70 Å between the two diagonally opposite sites. Interestingly, the distance between the beginnings of the two C-terminal helices (α7, the Rho-interacting region) of Psu is 60 Å, whereas it is 80 Å between the middle of α7 helices [(12) Supplementary Figure S7A)].

**Figure 6.**
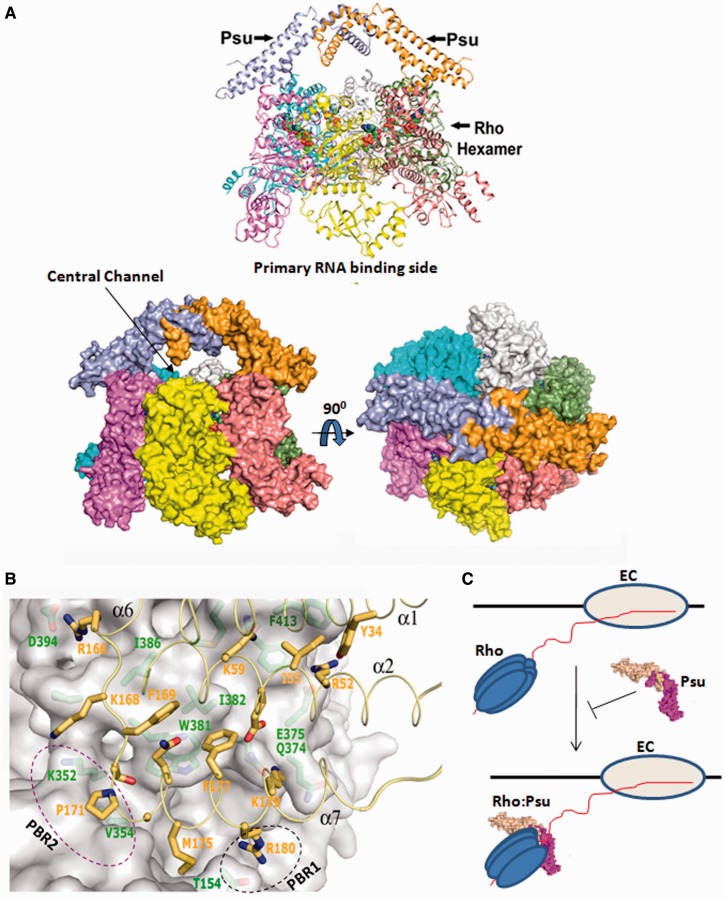
Structural modelling of Rho:Psu complex*.* (**A**) Above: cartoon representation of the docked model showing the orientation of Psu (gold and sky), when bound to the Rho hexamer (subunits are in magenta, yellow, salmon, green, grey and cyan in anti-clockwise manner). Below: surface representation of the same in two orientations. ATP, BeF3 and Mg^+2^ molecules are shown as spheres. Psu dimer occupies the regions near the two diagonally opposite ATP-binding sites of Rho and blocks its central channel. A 90° rotated view shows that the Psu dimer covers the central channel. (**B**) A close-up view showing the residues of Psu (yellow sticks) interacting with Rho (the surface and the green sticks), having the complementarities both in shape and chemistry. The residues of α7 of Psu are seen to be in close proximity to the region encompassing residues 151–155 (part of PBR1), and α6–α7 loop region is close to the residues, 350–354 (part of PBR2), of Rho. These regions are indicated by dotted ovals. (**C**) A cartoon showing the action of Psu as a polarity suppressor. The EC carrying a hexameric Rho on its nascent RNA is indicated. Psu dimer binds with the closed ring state of Rho, interacts near the ATP-binding site and inhibits the translocation of the latter on the nascent RNA.

We docked the Psu dimer on the Rho hexamer, and we refined the model (see the ‘Materials and Methods’ section) using the aforementioned information. During this modelling process, we maintained a symmetric nature of the interaction, and we assumed that the Psu-homodimer interacts with the Rho-homohexamer (Figure 6A and B). The binding free energy of the docked model is −7 Kcal/mol, which suggests an energetically favourable interaction. Other configurations, using the same interactions sites, were not energetically favourable.

This docking was performed without introducing major structural alterations in Psu and Rho. In this docked structure, a Psu dimer bridges the two diagonally opposite inter-subunit groove of the Rho hexamer (PDB code: 3ICE), located near the ATP and secondary RNA-binding sites (Figure 6A and B). This configuration seems to block the RNA exit path of Rho. There are three pairs of potential interaction sites on the Rho hexamer, capable of binding three Psu dimers. However, because of steric hindrance only one dimer of Psu can bind to a Rho hexamer at a time. The selection of the docking sites is likely to be random. Experimentally observed 2Psu:2Rho cross-linked species in Figure 4A and Supplementary Figure S4C resembles the modelled configuration of a dimer of Psu on a hexamer of Rho.

Figure 6B demonstrates a close-up view of the Rho–Psu interaction surface. The shape and the chemical complementarities of the docked model are evident at several points. Psu makes most of the interactions with Rho through its α6–α7 loop and α7 helix. The region, Thr 168-Ala 172 (α6–α7 loop) of Psu occupies a cleft on the surface of Rho, encompassing the residues 348–354 (PBR2). α7 of Psu is placed close to a shallow groove on the surface of Rho, surrounded by the residues 149–154 (PBR1), indicating that this region of Rho might be involved in direct interactions with Psu.

This configuration of Rho–Psu complex predicts that (i) Psu dimer will block the central hole of the Rho hexamer and (ii) its presence at the RNA exit channel will cause mechanical obstacle to the translocase activity of Rho (see the cartoon in Figure 6C). We validated these two predictions in the following sections.

### Psu makes the central channel of Rho less accessible

**Figure 7.**
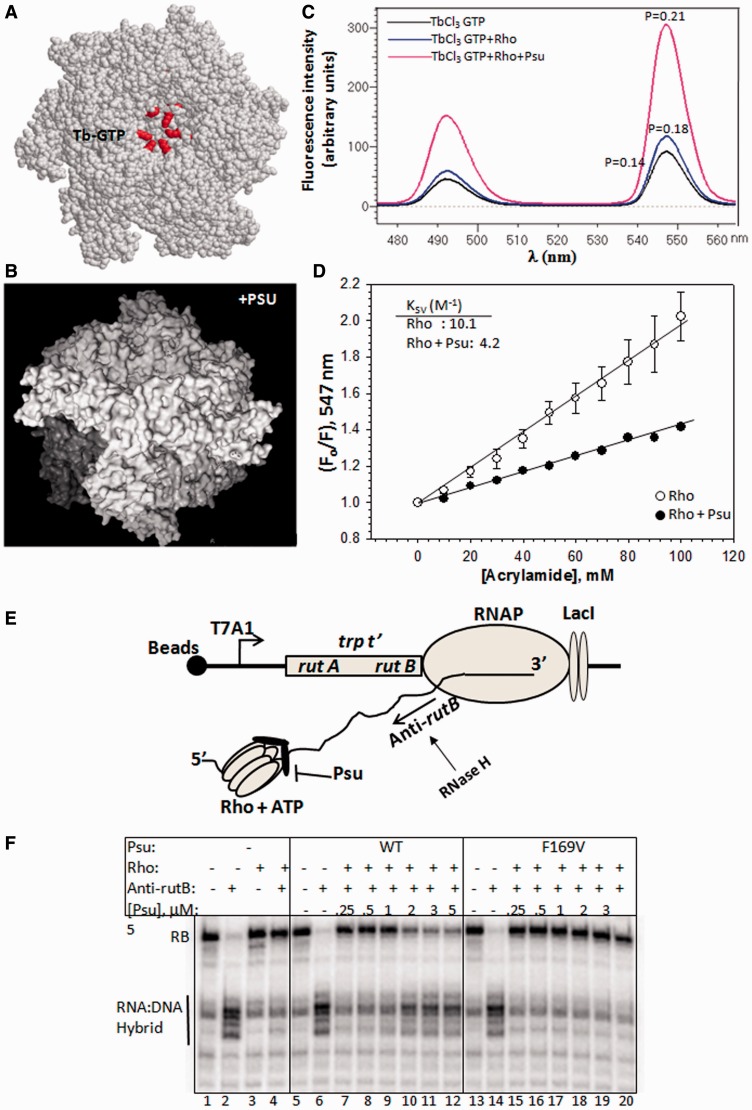
Accessibility of the central channel of Rho and blocking its translocase activity. (**A**) Rho hexamer showing the putative location of Tb-GTP-binding sites in the central channel. The ATP-binding amino acids are shown in red spheres, and assumed to interact also with the Tb-GTP (13). (**B**) Configuration of Psu (whitish) in Rho:Psu complex blocking the access of the central channel of Rho (grey). (**C**) Fluorescence emission spectra of Tb-GTP under different conditions as indicated. Fluorescence polarization (P) values of different species at 547 nm are also indicated. (**D**) Stern–Volmer plots to determine the quenching constant (K_SV_) and the nature of quenching by a neutral quencher, acrylamide. *F* and *F*_o_ are fluorescence intensities of Tb-GTP in the presence of different concentrations of acrylamide and the initial intensity, respectively. Average K_SV_ values are indicated. Errors were calculated from two to three measurements. (**E**) Cartoon showing the design of the experiment using a stalled EC downstream of the *trpt'* terminator. Site of RNA:DNA hybrid formation is indicated. (**F**) Autoradiogram showing the extent of cleavage of the RNA:DNA hybrid under different conditions as indicated. RB indicates the size of the RNA emerging out of the stalled EC.

A lid-like Psu dimer over the central channel of Rho (Figure 6) is predicted to make the channel less accessible to the solvent, and it could render the catalytic pocket less accessible to ATP molecules (Figure 7A and B). We probed the accessibility of this catalytic pocket by measuring the change in fluorescence intensity and quenching of a fluorescent analogue of GTP, Tb-GTP (a 3:1 complex of terbium chloride and GTP). Rho can use GTP instead of ATP, and presumably the Tb-GTP complex binds to the same site as ATP in the central channel (13). We observed a huge increase of fluorescence intensity of the Rho-bound Tb-GTP in the presence of Psu together with the increase in fluorescence polarization (Figure 7C), which indicates a Psu-induced burial of the catalytic pocket from the solvent. We next used fluorescence quenching technique to measure the accessibility of this Rho bound Tb-GTP to the solvent. Increasing concentration of acrylamide, a neutral quencher, was used to monitor the quenching of the fluorescence intensity (at 547 nm) of the Rho-bound Tb-GTP. The quenching constant (K_SV_, Stern–Volmer constant), a quantitative measure of the accessibility, was obtained from the plots of the fluorescence intensities against the acrylamide concentration (Figure 7D). The K_SV_ value reduced significantly when Psu was bound to Rho. This further supports the proposition that the central channel, as well as the catalytic pocket of Rho, is less accessible, when it is complexed with the Psu dimer.

### Psu renders mechanical impediment to the translocase activity of Rho

The configuration of Psu over the central channel of Rho (Figure 6) is likely to offer a physical obstacle to the helicase/translocase activity of Rho. This activity of the Rho protein, both in the presence and absence of Psu, was measured from the former’s potential to unwind an artificial RNA:DNA hybrid made on the nascent RNA of the EC, stalled at a lac operator site located downstream of the *trp t’* terminator [Figure 7E (22)]. This RNA:DNA hybrid was formed at the *rutB* site of this terminator that is located just upstream of the road-blocked EC (RB), and it is separated by >60 nt from the major Rho-loading site, *rutA*. A DNA-oligo, antisense to the rutB (anti-rutB) sequence, was used to form this artificial RNA:DNA hybrid. We have measured the RNA:DNA hybrid unwinding event by monitoring the sensitivity of the RNA in the hybrid to RNase H. Presence of a RNA:DNA hybrid makes this region sensitive to RNase H digestion (Figure 7F, lanes 2, 6 and 14). And this digestion was negligible, when the ATP-dependent unwinding of this hybrid occurred by the helicase/translocase activity of Rho (Figure 7F, lane 4). We observed that, this region of RNA was sensitive to RNase H under two conditions; when Rho was not added (lane 2), and in the presence of higher concentrations of Psu (lanes 10–12). This result suggests that Rho is unable to unwind the RNA:DNA hybrids at higher concentration of Psu. This effect of Psu was due to the specific Rho–Psu complex formation because a Psu mutant, F169V, did not block the unwinding activity of Rho (lanes 15–20). Hence, we concluded that the formation of a ‘Psu-lid’ over the central channel of Rho renders mechanical impediment to its translocation motion along the RNA.

## DISCUSSION

Bacteriophage P4 capsid protein, Psu, anti-terminates the well-conserved transcription termination function of Rho (8–10). In this study, we described the interaction surface, the configuration of the Rho–Psu complex and the structural and mechanistic basis of the Psu-induced anti-termination process. We provided the following evidence. (i) We isolated and characterized two suppressors on Rho that specifically rectified the functional defects of the Psu mutants residing in its C-terminal Rho-binding region (Figures 1 and 2). (ii) Using protein–foot-printing, cross-linking and mutational analyses, we defined two regions, residues encompassing 149–153 (PBR1) and 347–354 (PBR2) of Rho, as the Psu-docking sites (Figures 3–5). One of the suppressors, N151D, is located in these binding sites. (iii) A molecular docking based on the symmetries and the structures of Rho and Psu, configuration of Psu on the P4 capsid structure, and the aforementioned experimental results, revealed that a V-shaped Psu dimer forms a ‘lid’ over the central channel of Rho (Figure 6). This conformation of the Rho–Psu complex provides the structural and mechanistic basis of Psu-induced inhibition of ATP binding and hydrolysis (10), and imposition of mechanical impediments to the translocase activity of Rho along the nascent RNA (Figure 7 and cartoon in Figure 6C).

A normal mode analysis of the Psu structure revealed that the V-shaped Psu dimer acts as an arm, where its knotted region resembles an elbow, allowing restricted degrees of freedom to the dimeric-interface, but a higher flexibility to its C-terminal tails [Supplementary Figure S7A (12)]. Interestingly, the same dimeric scaffold is used to recognize two unrelated hexameric proteins, Sid of the P4 capsid and the transcription terminator, Rho. The predicted dynamics of the C-terminal tail could play important role in using similar mode of binding to these different proteins. Even though the rigid-body docking of Psu on Rho is consistent with the experimental data, we cannot rule out the Psu-induced conformational changes of Rho. It should also be noted that the distance between these two helices of the Psu dimer is compatible with the dimension of the central channel of the ‘closed’ state hexamer of Rho [PDB: 2HT1 and 3ICE (23,24)]; hence, it is possible that Psu targets the translocating Rho.

Both Psu and the antibiotic, bicyclomycin (BCM), affect the ATP binding and the hydrolysis functions of Rho (25,26). Even though the outcome of their action is same, and their interaction sites are in close proximity (Supplementary Figure S7C), the mechanistic basis of their inhibition processes is different. BCM, being a small molecule, enters deep into the central channel of Rho and acts as a competitive inhibitor of the latter’s ATPase activity (27), whereas Psu, on binding to the outer rims of this channel (Figure 6A), exerts the same effect most likely by distorting the conformations of the central channel.

The hexameric RNA chaperon, Hfq, has been shown to bind and inhibit Rho by affecting the latter’s ATPase, as well as translocase activity (28) and, hence, functions in a similar manner as Psu. According to the symmetry of the Hfq-hexamer, it may bind at the rim of the RNA exiting side of the central channel of Rho, forming a ‘washer-like’ configuration. In this regard, it is likely that both Psu and Hfq may compete with each other for the same region of the Rho molecule *in vivo*.

*Will Psu inhibit Rho proteins from pathogenic bacteria?* The homology models of different Rho proteins from a diverse set of bacterial pathogens revealed that the looped out primary docking site of Psu is well conserved, so do their hexameric structures (Supplementary Figure S8). These make it likely that Psu will be capable of inhibiting Rho proteins from pathogens. As this conserved looped out region of Rho directly interacts with the terminal helix 7 of Psu, we speculate that peptide inhibitor(s) can be designed from the C-terminal regions of this P4 capsid protein.

## SUPPLEMENTARY DATA

Supplementary Data are available at NAR Online: Supplementary Table 1, Supplementary Figures 1–8 and Supplementary Methods.

## FUNDING

DST Swarnajayanti Fellowship (to R.S.); Center of Excellence on ‘Microbial Physiology’ of the Department of Biotechnology, Government of India. Laboratory of U.S. is supported by the intra-mural funding of Saha Institute of Nuclear Physics. Funding for open access charge: Waived by Oxford University press.

*Conflict of interest statement.* None declared.

## Supplementary Material

Supplementary Data
