# Supplementary material for: Structural and mechanistic basis of anti-termination of Rho-dependent transcription termination by bacteriophage P4 capsid protein Psu
Source: Nucleic Acids Res. 2013 May 22;41(14):6839–56. doi: 10.1093/nar/gkt336 (PMC3737525; doi:10.1093/nar/gkt336)
Supplement: Supplementary Data [file supp_41_14_6839__index.html]

Structural and mechanistic basis of anti-termination of Rho-dependent transcription termination by bacteriophage P4 capsid protein Psu — Structural and mechanistic basis of anti-termination of Rho-dependent transcription termination by bacteriophage P4 capsid protein Psu — Supplementary Data 

# Structural and mechanistic basis of anti-termination of Rho-dependent transcription termination by bacteriophage P4 capsid protein Psu

## Supplementary Data

files

**Files in this Data Supplement:**

- Supplementary Data - pdf file
