# Supplementary material for: Structural and mechanistic basis of anti-termination of Rho-dependent transcription termination by bacteriophage P4 capsid protein Psu
Source: Nucleic Acids Res. 2013 May 22;41(14):6839–56. doi: 10.1093/nar/gkt336 (PMC3737525; doi:10.1093/nar/gkt336)
Supplement: Supplementary Data [file supp_gkt336_nar-00817-v-2013-File009.pdf]

Supplementary Methods.

In vitro pull-down assays.

For *in vitro* pull-down assays (Figures S2 and S3A), 20 µg of the His-tagged Psu and 7 µg of the non-His-tagged Rho were mixed in 100 µl of binding buffer (pH 8.0) containing 100 mM NaH_2PO_4, 100 mM NaCl, 10 mM imidazole, and PMSF, and incubated at 37 °C for 10 min. Protein mixture was added to 100 µl of pre-equilibrated Ni-NTA beads, and incubated at room temperature for 10 min. The supernatant was removed after spinning at 2000 rpm for 2 min. The beads were then washed with 100 µl of wash buffer (100 mM NaH_2PO_4, 100 mM NaCl, and 50 mM imidazole), and the proteins were eluted with 100 µl of elution buffer composed of 100 mM NaH_2PO_4, 100 mM NaCl, and 500 mM imidazole. Samples were loaded on 12% SDS-PAGE.

Western blots analyses.

After running the SDS-PAGE, the proteins were transferred to PVDF membrane for western blot analysis. The blots were blocked in PBST buffer (10mM Na_2HPO_4, 1.76 mM KH_2PO_4, 137 mM NaCl, 2.7 mM KCl pH 7.4, 0.05% Tween-20(PBST)) plus 5% non-fat dry milk. The blots were washed three times for 10 minutes each with PBST. The blots were then incubated for 2 hrs in PBS with 1:60,000 dilution of Rho anti-sera and washed three times for 10 minutes each with PBST. They were subsequently incubated with 1:1,60,000 dilution of anti-rabbit horse radish peroxidase-conjugated secondary antibody (Sigma) in PBST for 1 hr at room temperature and washed as before. The blots were developed with the ECL (enhanced chemiluminescence) kit (GE healthcare) and exposed to the film.

CD spectroscopy.

CD spectroscopy of WT and different Rho mutants were performed using a JASCO 810 spectropolarimeter at 25 °C. The scanning was done from 250 to 200 nm in a cuvette with 0.1-

cm path length. The data pitch was at 1nm; bandwidth of 2 nm; response time of 2s, and scan speed of 50 nm/min were used for the measurements. Most of the recordings were done in the phosphate buffer (pH 7.0) [20 mM sodium phosphate, 100 mM NaCl]. The spectra of R144E and E148R mutants of Rho were recorded in buffer containing 20mM Tris-HCl (pH 8.0) and 100 mM NaCl. The estimation of secondary structure was done according to a published method (Yang, 1986), using the manufacturer's software for the instrument.

References:

Yang, J. T., Wu, C. S. & Martinez, H. M. (1986) Calculation of protein conformation from circular dichroism. *Methods Enzymol.*, **130**, 208–269.

Table S1. Strains, plasmids, and oligos.

Strains	Genotype	Reference
GJ3161	MC4100 *galEp3*	Harinarayanan and Gowrishankar, 2003
RS336	MC4100 galEp3 Δ*rho*::*Kan* with pHYD1201	Chalissery *et al.*, 2007
RS659	MG1655 Δ*rho::Kan* with pHYD1201	Chalissery *et al.*, 2007
RS1128	MC4100 *galEp3,* λRS45 lysogen carrying P_{lac}– H-19B *nutR-tR1- trpt'-lac ZYA,* Δ*rho::Kan* with pHYD1201	Shashni *et al.*, 2012
XL1-Red	*endA1 gyrA96 thi-1 hsdR17 supE44 relA1 lac mutD5 mutS mutT* Tn*10*Tet	Stratagene
XL1-Blue	recA1 endA1 gyrA96 thi-1 hsdR17 supE44 relA1 lac [F′ proAB $lacI^q$ZΔM15 Tn*10*Tet]	Stratagene
**Plasmids**	**Description**	
pHYD567	3.3 kb NsiI fragment carrying rho^+ cloned from λ phage 556 of Kohara library into PstI site of pCL1920 (pSC101; Sp^R, Sm^R)	Chalissery *et al.*, 2007
pHYD1201	3.3 kb HindIII-SalI fragment carrying rho^+ sub-cloned from pHYD567 into HindIII-SalI sites of pAM34 (pMB1; IPTG dependent replicon, Amp^R)	Harinarayanan and Gowrishankar, 2003
pRS96	WT *rho* cloned at NdeI/XhoI site of pET21b, His-tag at C-terminal; Amp^R	Chalissery *et al.*, 2007
pRS100	WT *rho* cloned at NdeI/XhoI site of pET21b, non-His-tagged; Amp^R	Pani *et al.*, 2006
pRS106	T7A1 promoter cloned at EcoRI/HindIII sites of pRS102; Amp^R	Pani *et al.*, 2006
pNL150	pGZ119EH bearing *psu*; Cm^R	Linderoth, 1997
pRS259	pNL150 with F169V *psu*; Cm^R	Linderoth, 1997
pRS343	pBAD18 vector; Amp^R	Guzman *et al.*, 1995
pRS370	pET33b with WT *psu* cloned at NdeI/XhoI site, HMK and His tag at N-terminal; Kan^R	Pani *et al.*, 2009
pRS554	pNL150 with P157S *psu*; Cm^R	Pani *et al.*, 2009
pRS555	pNL150 with E56K *psu*; Cm^R	Pani *et al.*, 2009
pRS556	pNL150 with L21P *psu*; Cm^R	Pani *et al.*, 2009
pRS557	pNL150 with S72L *psu*; Cm^R	Pani *et al.*, 2009
pRS558	pNL150 with R166C *psu*; Cm^R	Pani *et al.*, 2009
pRS563	pNL150 with R166P *psu*; Cm^R	Pani *et al.*, 2009
pRS564	pNL150 with P157L *psu*; Cm^R	Pani *et al.*, 2009
pRS569	pBAD18 with WT *psu* cloned at HindIII/EcoRI site; Amp^R	Pani *et al.*, 2009
pRS604	pTL61T with pT7A1-Lambda *nutR-tR1-T1T2-lacZYA*; Amp^R.	Pani *et al.*, 2009
pRS649	WT *rho* with its own promoter cloned at HindIII/SacI sites of pCL1920; $Spec^R$, $Strep^R$	Muteeb *et al.,*2012
pRS624	pET 28b with E56K *psu* cloned at NdeI/XhoI site, His-tag at N-terminal; Kan^R	Pani *et al.*, 2009
pRS627	pET28b with R166C *psu* cloned at NdeI/XhoI site, His-tag at N-terminal; Kan^R	Pani *et al.*, 2009
pRS633	pET28b with R166P *psu* cloned at NdeI/XhoI site, His-tag at N-terminal; Kan^R	Pani *et al.*, 2009
pRS790	pET33b with C117SC13S *psu*, cloned at NdeI/XhoI site, HMK/His tag at N-terminal; Kan^R	Pani *et al.*, 2009
pRS845	pET33b with S80C *psu*, cloned at NdeI/XhoI site, HMK/His tag at N-terminal; Kan^R	Pani *et al.*, 2009
pRS846	pET33b with S161C *psu*, cloned at NdeI/XhoI site, HMK/His tag at N-terminal; Kan^R	Pani *et al.*, 2009
pRS848	pET33b with S181C *psu*, cloned at NdeI/XhoI site, HMK/His tag at N-terminal; Kan^R	Pani *et al.*, 2009
pRS893	pCL 1920 with P167L *rho*; $Spec^R$, $Strep^R$	This study
pRS951	pET 21b with P167L *rho* cloned at NdeI/XhoI site, His-tag at C-terminal; Amp^R	This study
pRS961	pET 21b with C202S *rho* cloned at NdeI/XhoI site, His-tag at C-terminal; Amp^R	This study

pRS962	pET 21b with C202S *rho* cloned at NdeI/XhoI site, Non His-tag; Amp^R	This study
pRS963	pET 21b with P167L *rho* cloned at NdeI/XhoI site, HMKHis-tag at C-terminal; Amp^R	This study
pRS964	pET 21b with P167L *rho* cloned at NdeI/XhoI site, NonHis-tag; Amp^R	This study
pRS965	pET 21b with WT *rho* cloned at NdeI/XhoI site, HMKHis-tag at C-terminal; Amp^R	This study
pRS983	PCL 1920 with N151D *rho*; $Spec^R$, $Strep^R$	This study
pRS993	pET 21b with N151D *rho* cloned at NdeI/XhoI site, NonHis-tag; Amp^R	This study
pRS994	pET 21b with N151D *rho* cloned at NdeI/XhoI site, His-tag at C-terminal; Amp^R	This study
pRS995	pET 21b with N151D *rho* cloned at NdeI/XhoI site, HMKHis-tag at C-terminal; Amp^R	This study
pRS1020	pET 28b with P157L *psu* cloned at NdeI/XhoI site, His-tag at N-terminal; Kan^R	This study
pRS1198	pCL 1920 with R144E *rho*; $Spec^R$, $Strep^R$	This study
pRS1199	pCL 1920 with R146E *rho*; $Spec^R$, $Strep^R$	This study
pRS1200	pCL 1920 with E148R *rho*; $Spec^R$, $Strep^R$	This study
pRS1201	pCL 1920 with R149E *rho*; $Spec^R$, $Strep^R$	This study
pRS1216	pET 21b with R144E *rho* cloned at NdeI/XhoI site, NonHis-tag; Amp^R	This study
pRS1219	pET 21b with R146E *rho* cloned at NdeI/XhoI site, NonHis-tag; Amp^R	This study
pRS1222	pET 21b with E148R *rho* cloned at NdeI/XhoI site, NonHis-tag; Amp^R	This study
pRS1225	pET 21b with R149E *rho* cloned at NdeI/XhoI site, NonHis-tag; Amp^R	This study
pRS1268	pET 21b with Δ144-148 *rho*, NonHis-tag; Amp^R	This study
pRS1269	pCL 1920 with Δ144-148 *rho*; $Spec^R$, $Strep^R$	This study
pRS1318	pET 21b with P167L C202S *rho* cloned at NdeI/XhoI site His-tag at C-terminal; Amp^R	This study
pRS1319	pET 21b with P167L C202S S346C *rho* cloned at NdeI/XhoI site His-tag at C-terminal; Amp^R	This study
pRS1359	pET 21b with P167L C202S S153C *rho* cloned at NdeI/XhoI site His-tag at C-terminal; Amp^R	This study
pRS1370	pCL 1920 with K352A *rho*; $Spec^R$, $Strep^R$	This study
pRS1372	pCL 1920 with V354N *rho*; $Spec^R$, $Strep^R$	This study
pRS1373	pET 21b with K352A *rho* cloned at NdeI/XhoI site, NonHis-tag; Amp^R	This study
pRS1375	pET 21b with V354N *rho* cloned at NdeI/XhoI site, NonHis-tag; Amp^R	This study
pRS1376	pCL 1920 with Δ149-153 *rho*; $Spec^R$, $Strep^R$	This study
pRS1377	pET 21b with Δ149-153 *rho*, NonHis-tag; Amp^R	This study
**Oligos**		
RK1	CGCCAGGGTTTTCCCAGTCACGAC; RP in the *lacZ* gene of pTL61T.	
RS58	ATAAACTGCCAGGAATTGGGGATCG; FP of pTL61T (and all its derivatives like pRS106, pRS25) vector sequence.	
RS78	GCTAGTTATTGCTCAGCGGT; T7 terminator sequence	
RS79	TAATACGACTCACTATAGGG; T7 promoter sequence	
RS83	ATAAACTGCCAGGAATTGGGGATCG; 5' biotinylated RS58	
RS84new	GCG CGC GCC ATA TGA ATC TTA CCG AAT TAA AG; rho upstream oligo with NdeI site.	
RS85	GCG CGC CTC GAG_TTA TGA GCG TTT CAT CAT TTC; rho RP with XhoI site and with stop codon.	
RS85a	GCG CGC CTC GAG_TGA GCG TTT CAT CAT TTC; rho RP with XhoI site and without stop codon.	
RS147	GCGCGCGGATCCCCCCATTCAAGAACAGCAAGCAGC, lamdbda TR1 reverse primer with BamHI	
RS177	GAATTGTGAGCGCTCACAATTCGGATATATATTAACAATTACCTG; lacO fusion at 161U of trpt' terminator.	
RS404	GAATTGTGAGCGCTCACAATTCGGATGCCAGACCGCGC TGGGTAAGCG; RP with	

	lacO in H-19B tR1 termination region, used to make Road block template.	
RS530	CAACCACCCGGATAGCGTGCTGATGGTTCTGC; FP to make Rho C202S by SDM	
RS531	GCAGAACCATCAGCA CGCTATCCGGGTGGTTG; RP to make Rho C202S by SDM	
RS707	GCT GCA CGC AAA CTC TGA GCT GCG TAT GGA ACG TGG; FP to make R144E of Rho by SDM.	
RS708	CCA CGT TCC ATA CGC AGC TCA GAG TTT GCG TGC AGC; RP to make R144E of Rho by SDM	
RS709	CGC AAA CTC TCG TCT GGA GAT GGA ACG TGG TAA CGG ; FP to make R146E of Rho by SDM	
RS710	CCG TTA CCA CGT TCC ATC TCC AGA CGA GAG TTT GCG ; RP to make R146E of Rho by SDM	
RS711	CTC TCG TCT GCG TAT GCG ACG TGG TAA CGG TTC TAC; FP to make E148R of Rho by SDM	
RS712	GTA GAA CCG TTA CCA CGT CGC ATA CGC AGA CGA GAG; RP to make E148R of Rho by SDM	
RS713	CGT CTG CGT ATG GAA GAG GGT AAC GGT TCT ACT G; FP to make R149E of Rho by SDM	
RS714	CAG TAG AAC CGT TAC CCT CTT CCA TAC GCA GAC G; RP to make R149E of Rho by SDM	
RS769	GTT ACC ACG AGA GTT TGC GTG CAG CGG GGT TAA GTT CTC AAA G; RP to make 144-148 a. a Δ (deletion) in WT Rho with 18 mer overhang region	
RS770	GCA AAC TCT CGT GGT AAC GGT TCT ACT GAA GAT TTA ACT GCT C;FP to make 144-148 a. a Δ (deletion) in WT Rho with 18 mer overhang region	
RS773	CGT ATG GAA ACT GAA GAT TTA ACT GCT CGC GTA CTG GAT CTG G; FP for 149-153 a. a Δ (deletion) in WT Rho with 18 mer overhang region	
RS774	ATC TTC AGT TTC CAT ACG CAG ACG AGA GTT TGC GTG CAG CGG G; RP for 149-153 a. a Δ (deletion) in WT Rho with 18 mer overhang region	
RS789	GGC AAC ATG GAA CTG CAC CTC TGT CGT AAG ATC GCT GAA AAA CGC; FP to make S346C by SDM on WT Rho	
RS790	GCG TTT TTC AGC GAT CTT ACG ACA GAG GTG CAG TTC CAT GTT GCC; RP to make S346C by SDM on WT Rho.	
RS791	CGT AAG ATC GCT GAA GCA CGC GTC TTC CCG GC; FP to make K352A by SDM on WT Rho	
RS792	GCC GGG AAG ACG CGT GCT TCA GCG ATC TTA CG;RP to make K352A by SDM on WT Rho	
RS795	GAT CGC TGA AAA ACG CAA CTT CCC GGC TAT CGA C;FP to make V354N by SDM on WT Rho	
RS796	GTC GAT AGC CGG GAA GTT GCG TTT TTC AGC GAT C; RP to make V354N by SDM on WT Rho	

References:

1. Harinarayanan, R., and Gowrishankar, J. (2003) Host factor titration by chromosomal R-loops as a mechanism for runaway plasmid replication in transcription termination defective mutants of Escherichia coli. *J. Mol. Biol.*, **332**, 31-46.

2. Sashni,R., Mishra,S., Kalyani, B.S., and Sen,R. (2012) Suppression of in vivo Rho dependent transcription termination defects: evidence for kinetically controlled steps. *Microbiology,* **158**, 1468- 1481.

3. Muteeb, G., Dey, D., Mishra, S., and Sen, R. (2012) A multi-pronged strategy by an antiterminator to overcome Rho-dependent termination. *Nucleic Acids Research,* **40(22)**, 11213-11228.

4. Guzman, L. M., Belin, D., Carson, M. J., and Beckwith, J. (1995) Tight regulation, modulation, and high-level expression by vectors containing the arabinose PBAD promoter. J. *Bact.*, **177 (14)**, 4121-4130.

5. Linderoth, N. A., Tang, G., and Calendar, R. (1997). In vivo and in vitro evidence for an anti-Rho activity induced by the phage P4 polarity suppressor protein Psu. *Virology*, **227**, 131-141.

MESTALQQAFDTCQNNKAAW ***L***(21)QRKNELAAAEQEYLRLLSG EGRNVSRLDELRNII***E***(56)VRKW

QVNQAAGRYIR***S***(72)HEAVQHIS IRDRLNDFMQQHGTALAAAL APELMGYSELTAIARNCAIQ

RATDALREALLSWLAKGEKI NYSAQDSDILTTIGFR***P***(157)DVA SVDDS***R***(166)EKFT PAQNMIFSRK

SAQLASRQSV

Figure S1A: Point mutations of Psu. Location of the point (in bold italics) mutations on the primary sequence of Psu. The C-terminal 10 and 20 amino acid deletions are indicated by rectangular boxes (11).

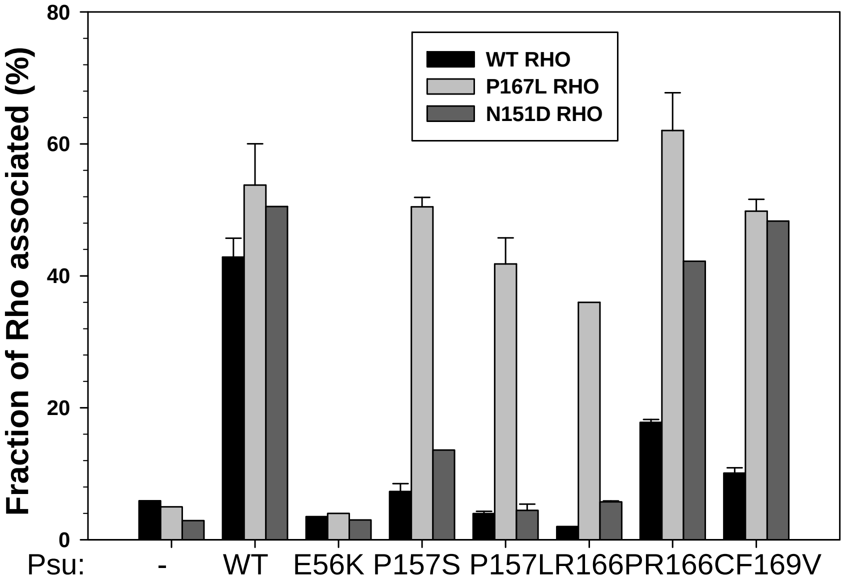

Figure S1B: Quantitative assesments of the pull-down assays described in figure 2A, B. Bar diagrams showing the amount of Rho eluted with different Psu mutants. Fraction of Rho associated (%) was calculated as {[E]/ ([FT]+[W]+[E])}. Error bars were calculated from two to three independent measurements.

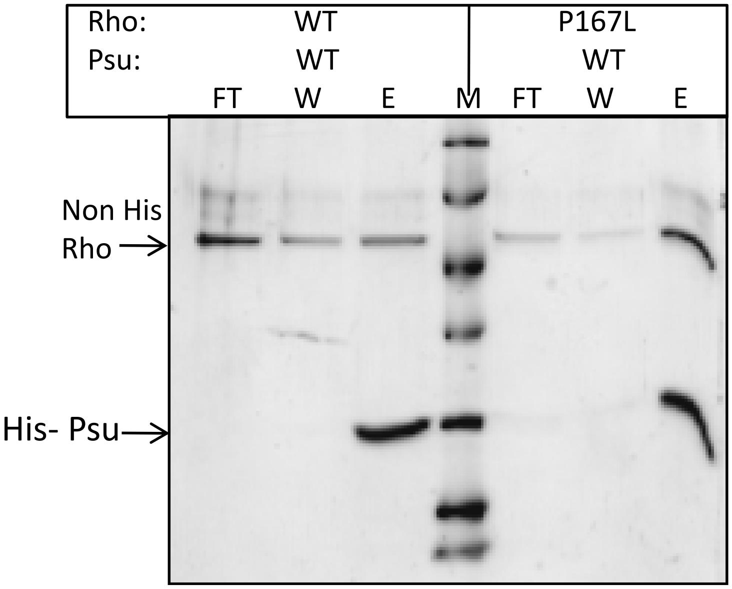

Figure S2: **In vitro stable binding of Psu and P167L Rho used in cross-linking and foot-printing assays of figures 3 and 4***.* *In vitro* pull-down assays of WT and P167L Rho with his-tagged WT Psu in the presence of 1 mM ATP. Rho-Psu mixture was passed through Ni-NTA beads and flow-through (FT), wash (W) and elute (E) fractions were collected. Stable complex was observed to be formed with Rho hexamer: Psu dimer:: 1:3. This indicates the presence of ~30% active Psu molecules.

Single cys derivatives are capable of forming Rho: Psu complex

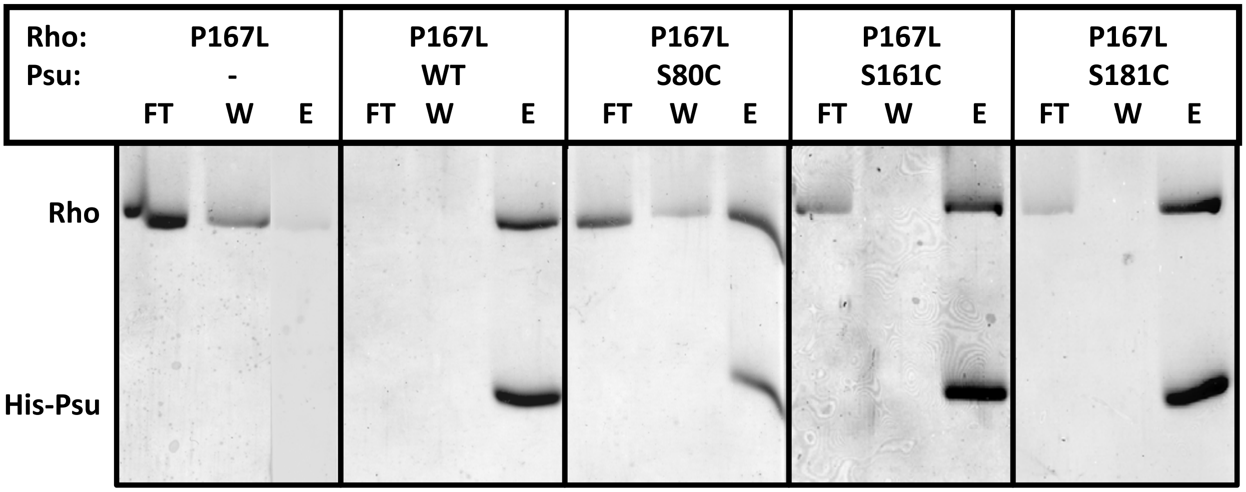

Figure S3A : *In vitro* pull-down assays of P167L Rho with his-tagged WT and single cys Psu derivatives in the presence of 1 mM ATP. Rho-Psu mixture was passed through Ni-NTA beads and flow-through (FT), wash (W) and elute (E) fractions were collected. Stable complex was observed to be formed with Rho hexamer: Psu dimer:: 1:3.

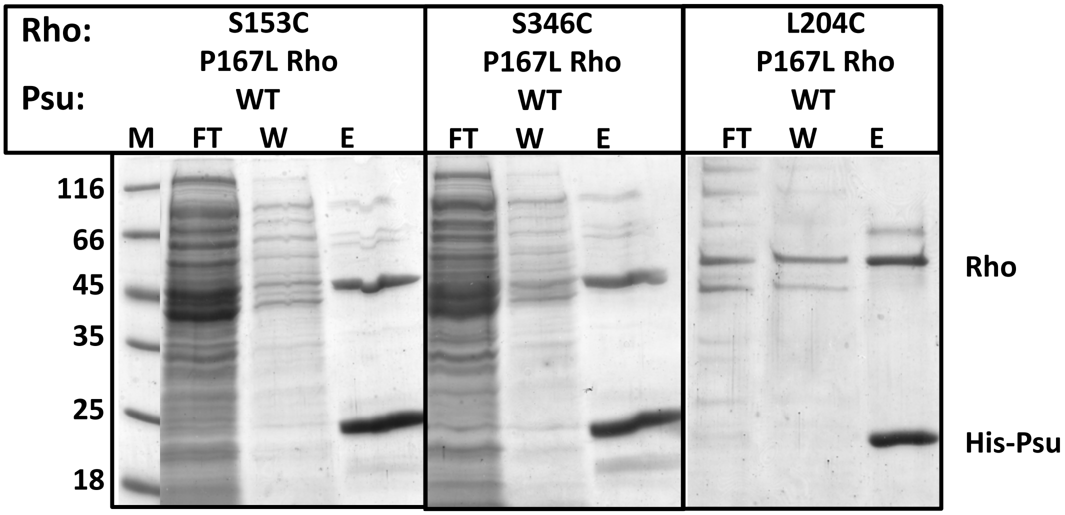

Figure S3B: *In vivo* complex formation between different derivatives of Rho and Psu proteins. Both His-tagged Psu and Non His-tagged Rho proteins were over-expressed from two different plasmids and cell-lysate was passed through the Ni-NTA beads. Flow through (FT) and wash (W) fractions contain the unbound Rho, whereas amount of protein in elute (E) fraction gave the measure of Psu-bound Rho. Both the proteins bands are indicated.

<u>**SPDP crosslinking (figure 4A in the text)**</u>

A)

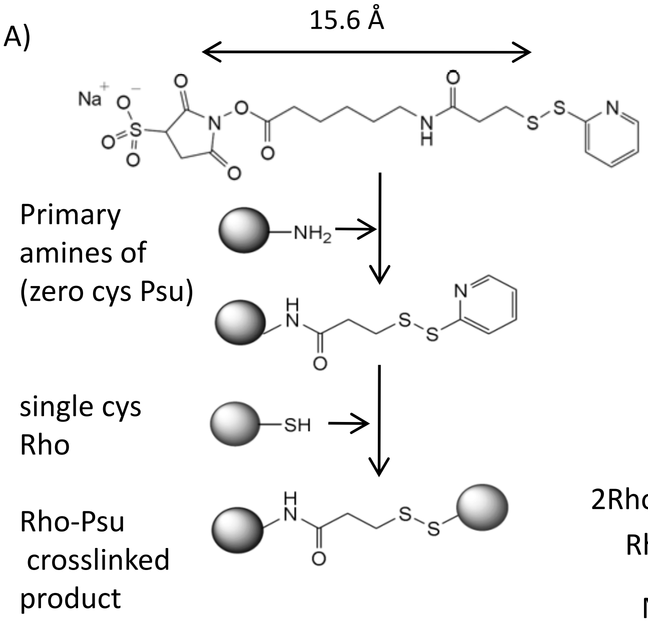

Figure S4A: Conjugation process of Bi-functional crosslinker LC-SPDP (sulfosuccinimidyl 6-[3'(2-pyridyldithio)-propionamido] Hexanoate)

B)

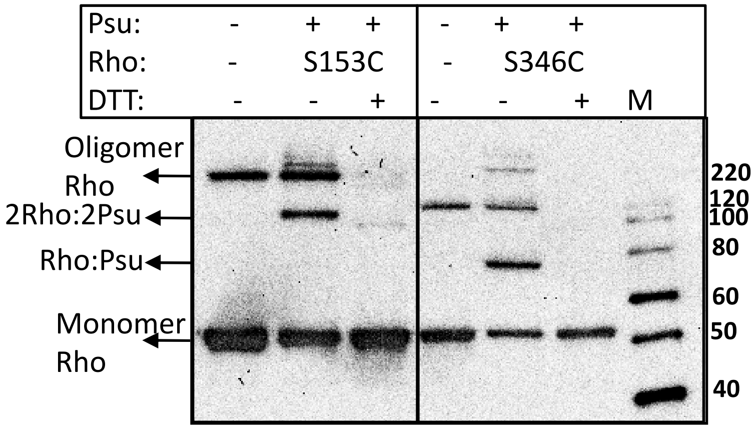

Figure S4B: The western blot of the SDS PAGE similar to that shown in figure 4A using a Polyclonal antibody of Rho. Different species are indicated.

C)

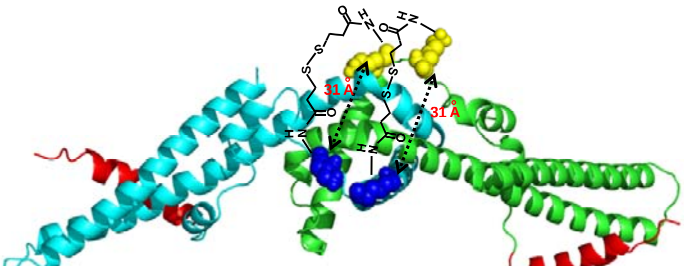

Psu-S-S-Psu

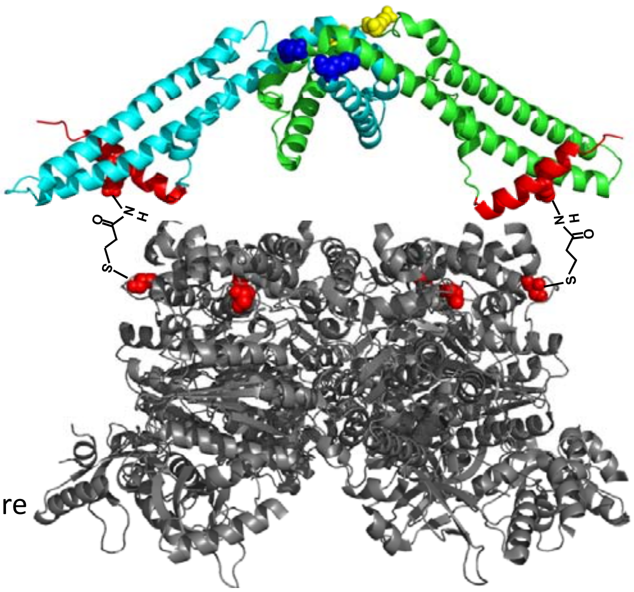

2Psu:2Rho

Figure S4C. Possible Intra-molecular dimerization of Psu formed by S-S linkages between two SPDP adducts from the two subunits. This can form if two lysines are located within ~30 Å. Dimer of Psu thus formed may also X-link with two cys residues from two subunits of Rho. In this case free SPDP adducts (near the helix 7) will participate in X-linking with the cys residues of rho. Cys residues on Rho are indicated as red sphere. Helix 7 of Psu is shown as red helix.

A)

Checking the conformational changes of the Rho mutants

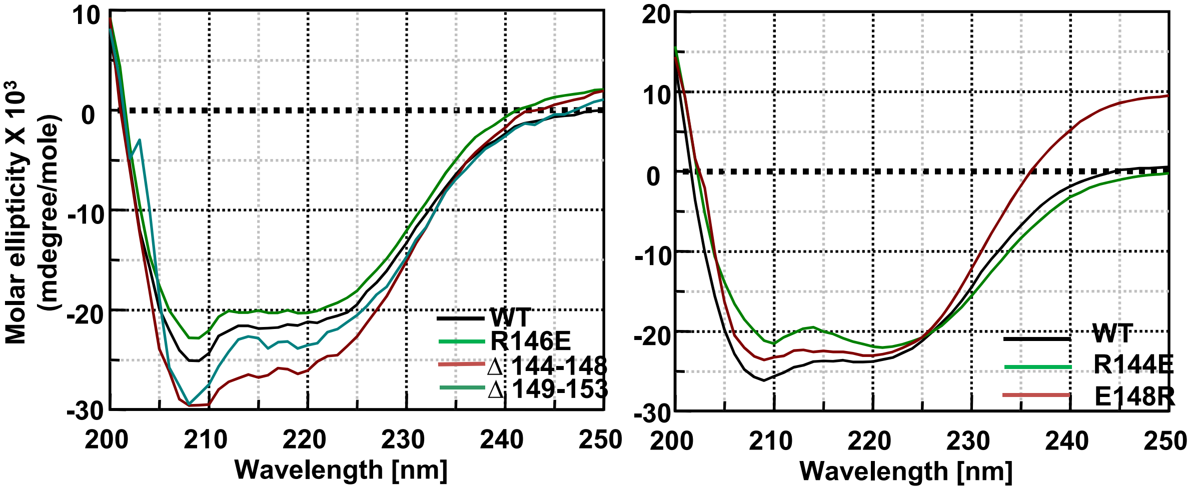

Secondary structure composition (%)	WT Rho	R146E Rho	Δ 144-148 Rho	Δ 149-153 Rho
α-Helix	27.0	29.3	25.5	28.4
β- Sheet	25.8	23.1	25.5	14.6
Turn	17.6	17.5	18.5	26.1
Random coil	29.7	30.2	30.5	30.8

WT Rho (tris buffer)	R144E Rho	E148R Rho
31.0	30.2	33.2
29.8	24.3	10.5
13.8	22.3	21.8
25.4	23.2	34.5

Figure S5A: Circular dichroism spectra of indicated Rho mutants with secondary structure analyses. Concentration of proteins were ~ 0.3 - 0.5 mg/ml .

Checking the non-specific adsorption of the non-His tagged Rho derivatives

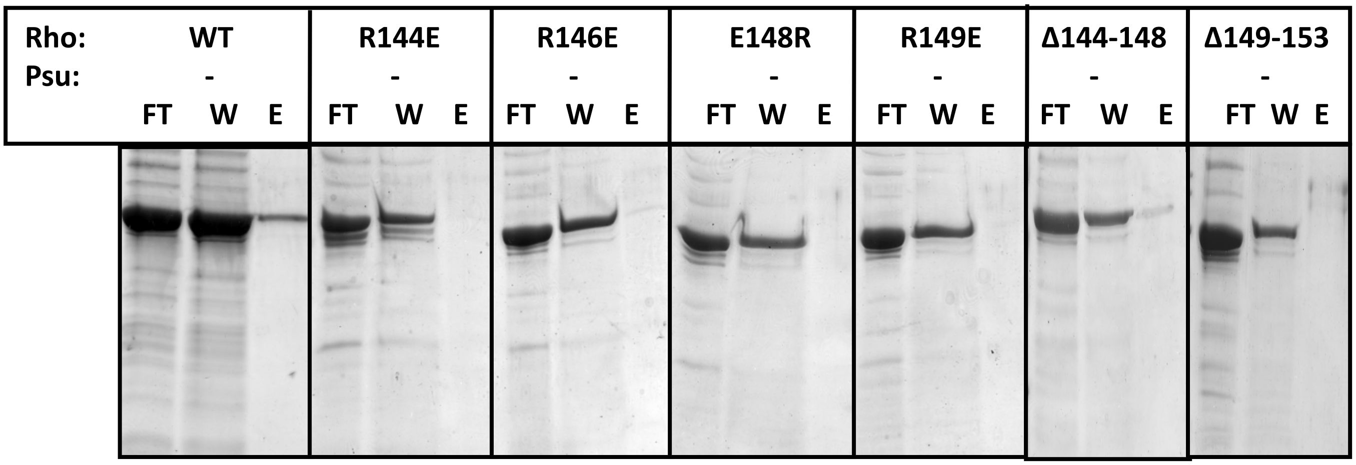

Figure S5B. Non-specific adsorption of the non-His tagged Rho mutants to the Ni-NTA beads. Non His-tagged Rho proteins were over-expressed and cell-lysate was passed through the Ni-NTA beads. Flow through (FT) and wash (W) fractions contain the unbound Rho whereas amount of protein in elute (E) fraction gave the measure of Rho bind to Ni-NTA beads. No Psu was present.

In vivo termination assays of different Rho mutants

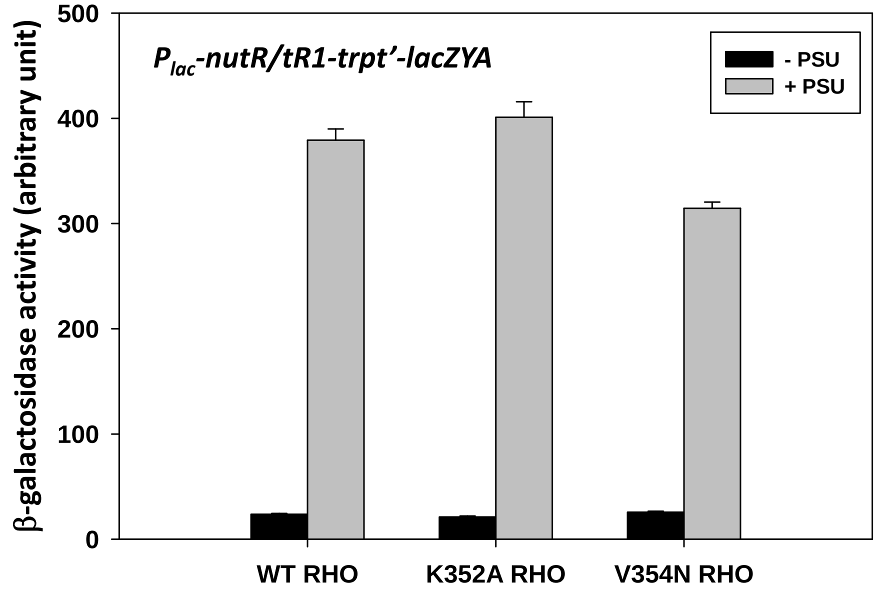

Figure S6A: *In vivo* antitermination assays of Rho activity in the absence and presence of Psu. Experiments were done in the same way as in figure 5D.

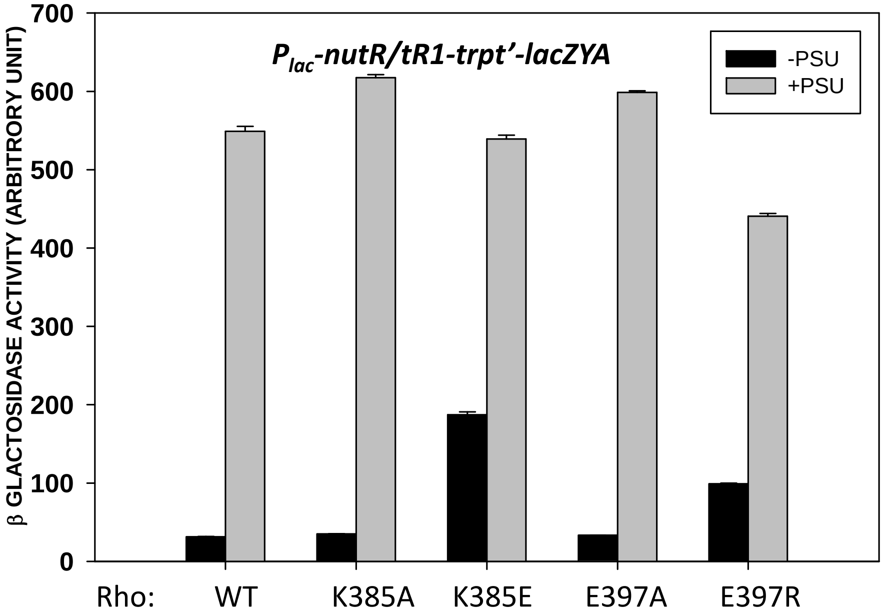

Figure S6B: *In vivo* antitermination assays of different Rho mutants. The values of β-galactosidase activity from the *lacZYA* reporter fused downstream of a double terminator (*nutR/tR1-trpt'*) cassette is plotted. Experiments were done in the same way as in figure S6A.

Structures of Rho and Psu

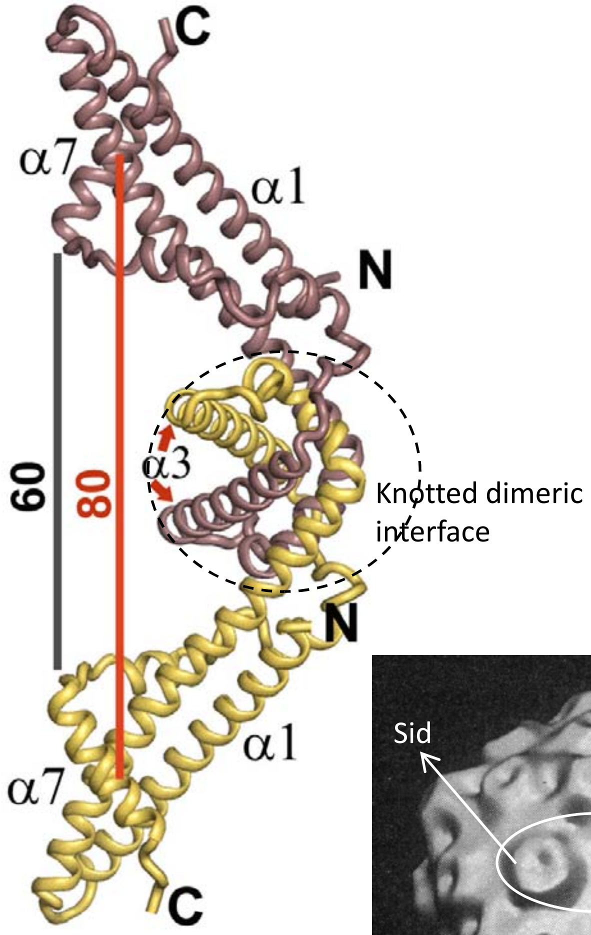

Figure S7A: The V shaped dimeric crystal structure of Psu (Banerjee et al., 2012). Each of the subunits are shown in violet and gold. Different structural elements are indicated. Distances between the two Rho-binding α7 helices are indicated.

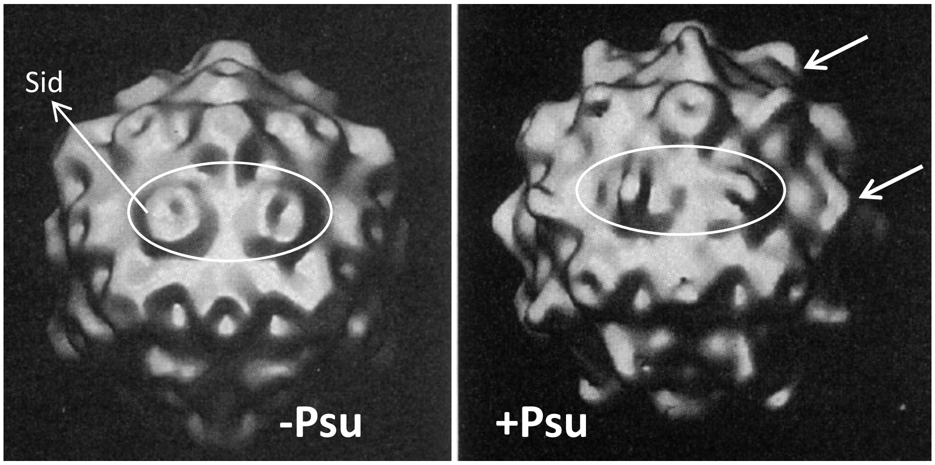

Figure S7B: The cryo-electron microgram of P4 capsid. The hexameric Sid proteins are indicated. V-shaped Psu proteins covering the central channel of Sid are also visible in +Psu panel (Dokland *et al*. 1993).

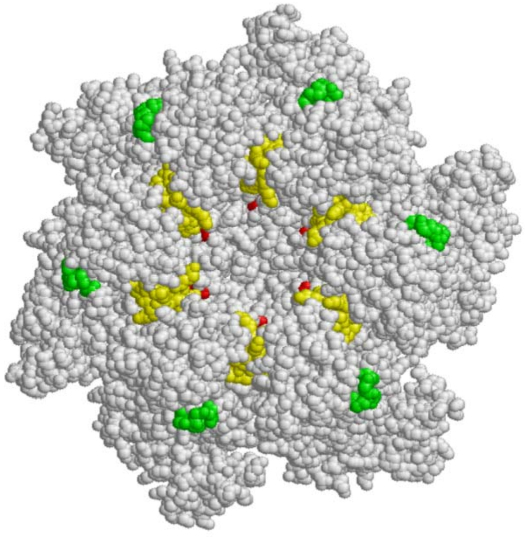

Figure S7C: The Bicyclomycin binding site inside the Channel (red spheres) is shown to be located near the PBR2 (yellow). PBR1 is in green.

Will Psu be an effective inhibitor of the Rho proteins from different pathogens?

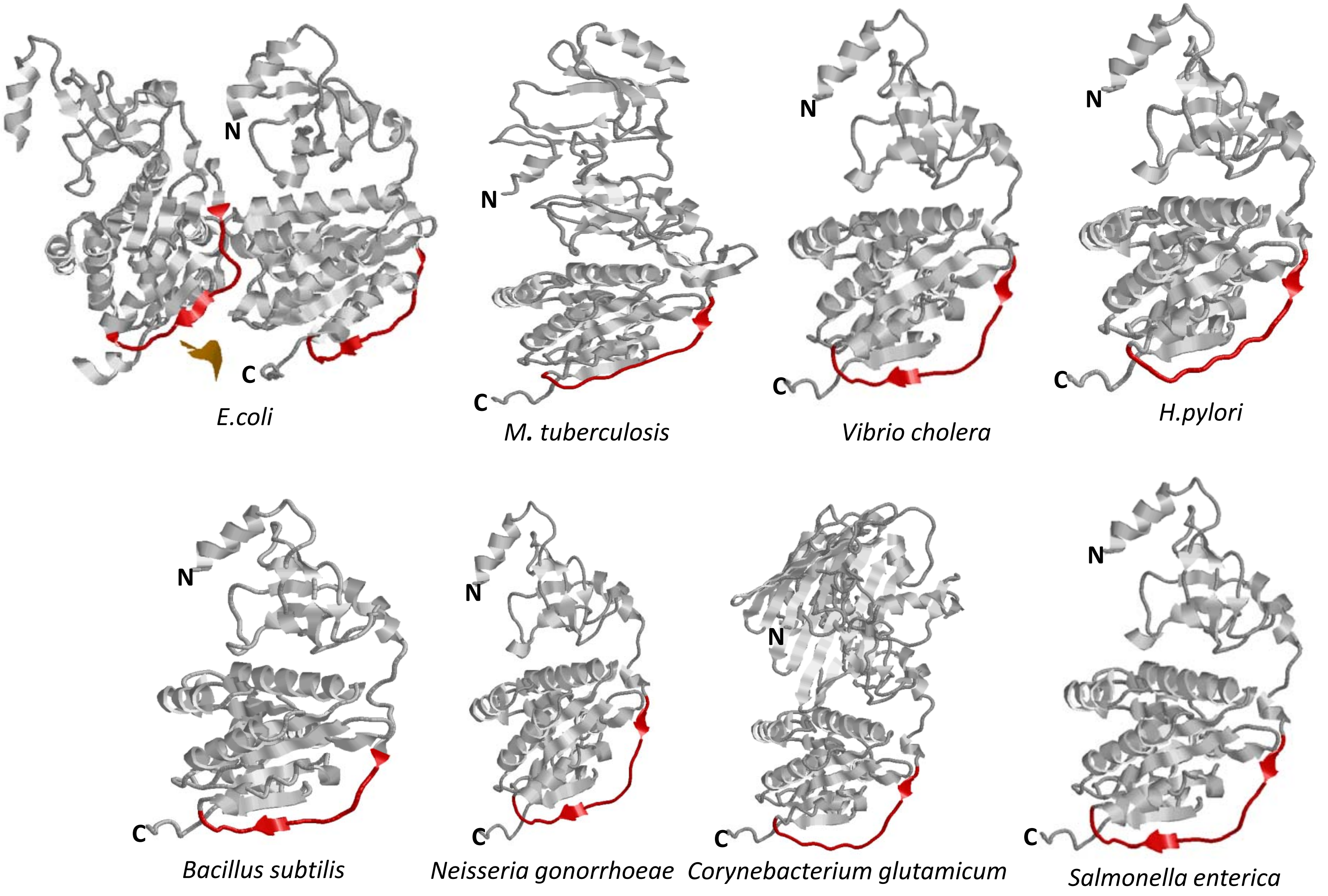

Figure S8: The homology models of Rho monomers from different pathogenic bacteria. The Psu-binding loop is conserved in many Rho proteins (shown in red).
